# Secondary Metabolites with Herbicidal and Antifungal Activities from Marine-Derived Fungus *Alternaria iridiaustralis*

**DOI:** 10.3390/jof9070716

**Published:** 2023-06-29

**Authors:** Jinqing Fan, Fangfang Guo, Chen Zhao, Hong Li, Tianli Qu, Lin Xiao, Fengyu Du

**Affiliations:** 1 College of Plant Health and Medicine, Qingdao Agricultural University, Qingdao 266109, China; fjq2831662399@163.com (J.F.); gff5172022@163.com (F.G.); zc171703121@163.com (C.Z.); li15145880540@163.com (H.L.); 2 College of Chemistry and Pharmacy, Qingdao Agricultural University, Qingdao 266109, China; qtli0411@163.com; 3 Shandong Key Laboratory of Applied Mycology, Qingdao Agricultural University, Qingdao 266109, China

**Keywords:** *Alternaria iridiaustralis*, agricultural bioactive potential, secondary metabolites, herbicidal activity, antifungal activity

## Abstract

Weed and soil-borne pathogens could synergistically affect vegetable growth and result in serious losses. Investigation of agricultural bioactive metabolites from marine-derived fungus *Alternaria iridiaustralis* yielded polyketides (**1**–**4**), benzopyrones (**5**–**7**), meroterpenoid derivatives (**8**), and alkaloid (**9**). The structures and absolute configurations of new **1**, **3**, **5**–**6,** and **8** were elucidated by extensive spectroscopic analyses, as well as comparisons between measured and calculated ECD and ^13^C NMR data. Compounds **1**–**4**, **6,** and **9** showed herbicidal potentials against the radicle growth of *Echinochloa crusgalli* seedlings. Especially **9** exhibited inhibition rates over 90% at concentrations of 20 and 40 μg/mL, even better than the commonly used chemical herbicide acetochlor. Furthermore, **9** also performed a wide herbicidal spectrum against the malignant weeds *Digitaria sanguinalis*, *Portulaca oleracea*, and *Descurainia sophia*. Compounds **5**–**8** showed antifungal activities against carbendazim-resistant strains of *Botrytis cinerea*, with minimum inhibitory concentration (MIC) values ranging from 32 to 128 μg/mL, which were better than those of carbendazim (MIC = 256 μg/mL). Especially **6** exhibited integrated effects against both soil-borne pathogens and weed. Overall, marine-derived fungus *A. iridiaustralis*, which produces herbicidal and antifungal metabolites **1**–**9**, showed the potential for use as a microbial pesticide to control both weed and soil-borne pathogens.

## 1. Introduction

Weed seeds widely distributed in soil could compete for nutrients, moisture, and light with vegetables, while soil-borne pathogens could directly invade vegetable roots and further reinforce weed harm [1,2]. For example, *Echinochloa crusgalli* is the most destructive malignant weed in the rice field [3], while *Botrytis cinerea* and *Fusarium oxysporum* are seriously damaging soil-borne pathogens that cause gray mold and wilt diseases of vegetables, respectively [4]. Due to ongoing unrestricted applications of chemical pesticides, weed and soil-borne pathogens have gradually developed multiple resistances, and, especially, no chemical pesticides could control both weed and soil-borne pathogens [5,6]. Therefore, the search for integrated biocontrol alternatives is always in demand.

*Suaeda glauca*, a kind of salt-tolerant plant, mainly grows in coastal or intertidal zones [7]. Due to its internal and external high-salinity environments, *S. glauca* has been considered a potential source for various bioactive endophytes, which could produce different interesting secondary metabolites [8,9,10,11,12]. The endophytic genus of *Alternaria* is a ubiquitous group growing in diverse ecosystems, producing a broad array of secondary metabolites. These metabolites mainly include polyketides, nitrogen-containing compounds, quinones, terpenes, and so on [13,14]. Research on their bioactive potentials mainly focused on the pharmacological applications, such as anticancer, antibacterial, antioxidant, and enzyme inhibitory effects, but there were few reports on their agricultural bioactive potential [13,14,15,16].

**Figure 1:**
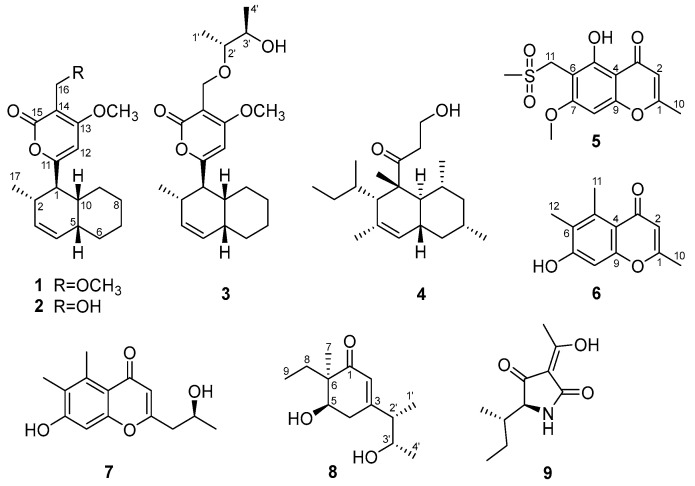
Chemical structures of isolated compounds **1**–**9**.

During our ongoing search for biocontrol agents in agriculture [8,9,10,17,18], *S. glauca*-derived endophytic fungus of *A. iridiaustralis* has attracted our attention because of its integrated potential against both weed and soil-borne pathogens. Our search for agricultural bioactive metabolites obtained nine ones (**1**–**9**), including four polyketides (**1**–**4**), three benzopyrones (**5**–**7**), one meroterpenoid derivative (**8**), and one alkaloid (**9**) (Figure 1). The isolation, structural elucidation, and agricultural bioactive evaluation of isolated metabolites are discussed herein.

## 2. Results and Discussion

### 2.1. Structure Elucidations

**Figure 2:**
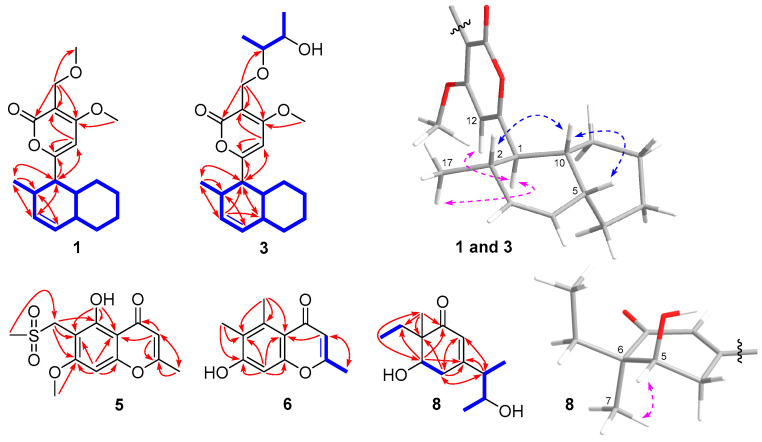
Key COSY (bond lines), HMBC (arrows), and NOE (dashed lines) correlations of **1**, **3**, **5**–**6** and **8**.

The molecular formula of compound **1** was obtained as C_19_H_26_O_4_ by HRESIMS (Figure S1 in the Supporting Information, SI), implying seven degrees of unsaturation. The one and two-dimensional NMR data (Table 1 and Figure 2) exhibited one carbonyl carbon (*δ*_C_ 167.5 CO) and three double bonds (*δ*_C_ 171.5 C, 170.8 C, 132.7 CH, 131.6 CH, 101.4 C, 98.6 CH), totally accounting for four degrees of unsaturation. Therefore, the remaining three degrees indicate the presence of three rings in the structure of **1**.

The decalin ring system, requiring two degrees of unsaturation, was deduced by the consecutive COSY cross-peaks from H-1 to H-10 and from H-2 to H_3_-17 (Figure 2), while the presence of one pyrone ring was confirmed by the observed HMBC correlations shown in Figure 2. Detailed analyses of its NMR data suggested that the structure of compound **1** was similar to that of solanapyrone B (compound **2**) [19], except the signals of one more OCH_3_ group (*δ*_H_ 3.33 and *δ*_C_ 58.3) were observed in ^1^H and ^13^C NMR spectra of **1**. The key HMBC correlation between H-16 and this OCH_3_ carbon further confirmed the linkage between C-16 and the OCH_3_ group (Figure 2).

Solanapyrone S (compound **3**) was confirmed to have the molecular formula C_22_H_32_O_5_ by its HRESIMS data, requiring seven degrees of unsaturation (Figure S9). Its one-dimensional NMR and HSQC data (Table 1 and Figure S13) exhibited marked similarities to those of solanapyrone B (compound **2**) [19], except the presence of 2′,3′-butanediol residue (CH_3_-1′ *δ*_H_ 1.29/*δ*_C_ 15.6, OCH-2′ *δ*_H_ 3.48/*δ*_C_ 81.0, OCH-3′ *δ*_H_ 3.79/*δ*_C_ 71.6, CH_3_-4′ *δ*_H_ 1.26/*δ*_C_ 18.4) was observed in ^1^H and ^13^C NMR spectra of **3**. Finally, the consecutive COSY cross-peaks from H_3_-1′ to H_3_-4′ and the key HMBC correlation between H-16 and OCH-2′ confirmed the connection between C-16 and 2′,3′-butanediol residue (Figure 2).

**Figure 3:**
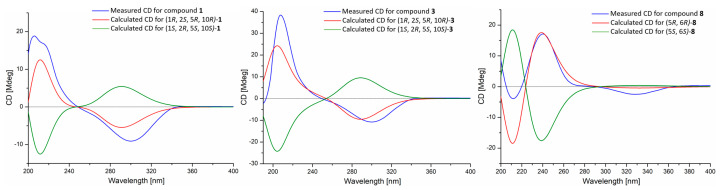
Comparisons of calculated ECD spectra with experimental ones of compounds **1**, **3** and **8** in CH_3_OH.

The NMR signals of **1** and **3** associated with the decalin unit were almost identical to those of solanapyrone B (**2**) [19], indicating their same relative configurations, which were confirmed by the key NOE correlations from H-10 to H-2 and H-5, as well as from H-1 to H-12 and H_3_-17 (Figure 2). The absolute configurations of decalin fragments of **1** and **3** were determined as 1*R*, 2*S*, 5*R,* and 10*R* via the agreement between the experimental and calculated ECD spectra, showing the same positive Cotton Effect (CE) around 210 nm and the negative CE near 295 nm (Figure 3). While the calculated ECD spectra of (1*S*, 2*R*, 5*S* and 10*S*)-**1** and **3** exhibited mirror-corresponding CEs. The same CEs of **1** and **3** should be related to their common pyrone and *cis*-decalin ring systems, while the 2′,3′-butanediol residue of **3** was far from the chromophore center and therefore did not exert the obvious effect of its CEs.

The DFT re-optimization of initial MMFF conformers of **1** and **3** at the B3LYP/6-311++g(d, p) level afforded three low-energy conformers above 1% population, respectively (Figures S36 and S37). Their further ^13^C NMR calculations could support the absolute configurations of the decalin fragments of **1** and **3** assigned by the ECD calculations and also confirm the 2′,3′-butanediol residue of **3** as 2′*R* and 3′*R* [20,21]. The correlation coefficients (*R*^2^) of **1** and **3** from linear regression analyses between calculated and experimental ^13^C NMR data were 0.9982 and 0.9979, respectively (Figure S39).

The HRESIMS data for compound **5** demonstrated its molecular formula to be C_13_H_14_O_6_S, indicating seven degrees of unsaturation (Figure S17). The one- and two-dimensional NMR data (Table 2 and Figure 2) exhibited one carbonyl carbon (*δ*_C_ 182.5 CO), six aromatic carbons (*δ*_C_ 164.0 C, 159.9 C, 158.6 C, 105.2 C, 101.2 C, 90.6 CH), and one double bond (*δ*_C_ 167.3 C, 109.3 CH), totally accounting for five degrees of unsaturation. Therefore, the remaining two degrees should be related to the cyclic ring systems.

Detailed analysis of the HMBC spectrum deduces the presence of a benzopyrone skeleton with CH_3_ (*δ*_H_ 2.39), OH (*δ*_H_ 13.35), and OCH_3_ (*δ*_H_ 3.95) groups substituted at C-1, C-5, and C-7, respectively (Figure 2). Considering its molecular formula and the representative fragmentation [M—SO_2_CH_3_]^+^ (219.06537), compound **5** should contain a SO_2_ group, which is rarely found in *Alternaria* metabolites (Figure S17). The one-dimensional NMR spectra of **5** showed remarkable similarities to those of chaetoquadrin D [22], except that signals of *N*-ethyl acetamide residue in chaetoquadrin D (*S*CH_2_ *δ*_H_ 3.20/*δ*_C_ 52.7, *N*CH_2_ *δ*_H_ 3.78/*δ*_C_ 32.8, CO *δ*_C_ 170.2, CH_3_ *δ*_H_ 1.94/*δ*_C_ 23.2) were absent from the NMR spectra of **5**. Instead, *S*CH_3_ signals (*δ*_H_ 2.91/*δ*_C_ 41.5) were observed, which was also confirmed by the key HMBC correlation between H-*S*CH_3_ to C-11 (Figure 2).

The molecular formula C_12_H_12_O_3_ of compound **6** was assigned on the basis of its HRESIMS data, indicating seven degrees of unsaturation (Figure S23). Detailed analyses of ^1^H-^1^H COSY and HMBC spectra confirmed the presence of a benzopyrone skeleton with three CH_3_ groups (*δ*_H_ 2.30, 2.75, and 2.17) substituted at C-1, C-5, and C-6, respectively (Figure 2). One-dimensional NMR spectra of **6** were almost identical to those of chaetosemin D (**7**) [23], except that signals of 2′-hydroxy propyl residue in **7** (CH_2_-1′ *δ*_H_ 2.69/*δ*_C_ 44.1, OCH-2′ *δ*_H_ 4.20/*δ*_C_ 66.9, CH_3_-3′ *δ*_H_ 1.29/*δ*_C_ 23.5) were absent from NMR spectra of **6**. Instead, CH_3_ signals (*δ*_H_ 2.30/*δ*_C_ 19.7) were observed in **6**.

Compound **8** showed the molecular formula C_13_H_22_O_3_ as determined by HRESIMS, suggesting three degrees of unsaturation (Figure S28). Its NMR data (Table 2 and Figure 2) exhibited one carbonyl carbon (*δ*_C_ 203.0 CO) and one double bond (*δ*_C_ 161.0 C, 126.1 CH), which indicated two degrees of unsaturation. Therefore, the remaining one degree should be related to the presence of one cyclic ring, which was also confirmed by the ^1^H-^1^H COSY and HMBC spectra (Figure 2). The ethyl and 3′-hydroxy butyl residues were deduced by the consecutive COSY cross-peaks from H_2_-8 to H_3_-9 and from H_3_-1′ to H_3_-4′. The key HMBC correlations from H_3_-7 to C-1, 6 and 8, as well as from H-2′ to C-2, 3 and 4, finally connected the ethyl and 3′-hydroxy butyl residues to C-6 and C-3, respectively.

The relative configuration of the cyclohexanone skeleton in compound **8** was deduced by the key NOE correlation from H-5 to H_3_-7 (Figure 2). The agreement of experimental and calculated ECD spectra of **8**, showing the same positive CE around 240 nm and negative CEs near 210 and 330 nm, confirmed the absolute configurations of the cyclohexanone fragment as 5*R* and 6*R* (Figure 3). Its further ^13^C NMR calculation deduced the absolute configuration of 3′-hydroxy butyl group as 2′*S* and 3′*S* [20,21]. The correlation coefficient (*R*^2^) of **8** from linear regression analysis between calculated and experimental ^13^C NMR data was 0.9948 (Figures S38 and S39).

The isolation of bioactive fractions from the culture extract also resulted in other known metabolites, including polyketides (**2** and **4**), benzopyrone (**7**), and alkaloids (**9**). Their structures were determined by detailed analyses of their spectroscopic data and comparisons with previously published reports as follows: solanapyrone B (**2**) [19], probetaenone I (**4**) [24], chaetosemin D (**7**) [23], and tenuazonic acid (**9**) [25]. All known metabolites (**2**, **4**, **7,** and **9**) were first isolated from the species of *A. iridiaustralis*.

### 2.2. Herbicidal and Antifungal Evaluations

The isolated metabolites (**1**–**9**) were evaluated for their herbicidal and antifungal activities. The herbicidal potential was assessed using the representative malignant weed *E. crusgalli*, while the antifungal activity was assessed using two groups of representative soil-borne pathogens: carbendazim-resistant isolates of *B. cinerea* from grape (BCG) and strawberry (BCS), as well as *F. oxysporum* strains of *F. oxysporum* f. sp. *cucumerinum* (FOC) and *F. oxysporum* f. sp. *Lycopersici* (FOL).

**Figure 4:**
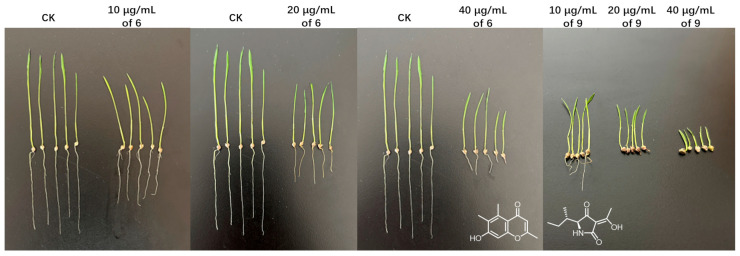
Herbicidal potentials of **6** and **9** against the growth of *E. crusgalli* seedlings.

The polyketides **1**–**4**, benzopyrone **6,** and alkaloid **9** showed herbicidal potentials against the radicle growth of *E. crusgalli* seedlings with a dose-dependent relationship (Table 3). Especially, **9** exhibited significant inhibition rates over 90% at concentrations of 20 and 40 μg/mL, even better than the commonly used chemical herbicide acetochlor, while **6** showed moderate inhibition rates of 60.3% and 72.6%, respectively (Table 3 and Figure 4). The further bioassay of the herbicidal spectrum of **9** suggested that it performed significant herbicidal potential against the malignant weed *Digitaria sanguinalis*, almost identical to that of acetochlor (Figure S40), while **9** also exhibited moderate activities against *Portulaca oleracea* and *Descurainia sophia* (Table S1). The preliminary structure-activity analysis of solanapyrone polyketides **1**–**3** indicated that the substituted group at C-16 should be related to their herbicidal activities.

Benzopyrones **5**–**6** and meroterpenoid derivative **8** showed antifungal potentials against two carbendazim-resistant strains of *B. cinerea* with MIC values ranging from 32 to 64 μg/mL, significantly better than those of carbendazim (MIC = 256 μg/mL) (Table 3). *B. cinerea* could widely invade various crops and vegetables during both the pre- and post-harvest stages. More seriously, its resistance to commonly used fungicides was developing year by year, also resulting in higher pesticide residue [4]. The antifungal target of carbendazim was related to *β*-tubulin proteins [26], suggesting that the antifungal mechanisms of **5**–**6** and **8** should be different from that of carbendazim. Furthermore, **6**–**8** also exhibited moderate antifungal activities against two *F. oxysporum* strains.

Alkaloid **9**, possessing a relatively simple skeleton and a wide herbicidal spectrum, showed the potential for use as a bio-herbicide. Although the antifungal and herbicidal activity of **6** was weaker than that of **8** and **9**, respectively, its integrated agricultural potential against both soil-borne pathogens and weeds indicated its application in the development of bio-pesticides.

## 3. Materials and Methods

### 3.1. General Procedures

NMR spectra were recorded at 500 and 125 MHz for ^1^H and ^13^C, respectively, on a Bruker Avance III spectrometer (Bruker, Rheinstetten, Germany). HRESIMS data were determined on a mass spectrometer of Thermo Scientific Orbitrap Fusion Lumos Tribrid (Thermo Scientific, MA, USA) and analyzed using Thermo Xcalibur 4.2 SP1. The circular dichroism (CD) spectrum was acquired on a JASCO J-810 CD spectrometer (JASCO, Tokyo, Japan). Column chromatography (CC) was performed with Silica gel (200–300 mesh; Qingdao Haiyang Chemical Co., Qingdao, China), Lobar LiChroprep RP-18 (40–63 μm; Merck, Kenilworth, NJ, USA), and Sephadex LH–20 (18–110 μm; Merck, Kenilworth, NJ, USA). Semi-preparative HPLC (semi-pHPLC) was performed using a Dionex HPLC system equipped with a P680 pump (flow rate: 3 mL/min), an ASI-100 automated sample injector, and a UVD340U multiple wavelength detector (Detection wavelength: 230 nm) controlled using Chromeleon software, version 6.80 (Dionex Corporation, Sunnyvale, CA, USA).

### 3.2. Fungal Strain and Weed Seeds

The fungal strain of *A. iridiaustralis* was isolated from the root of *S. glauca*, which was collected from the intertidal zone of the Yellow River Delta, Dongying, China, in October 2021. The fungus was identified on the basis of morphological characteristics and molecular analyses of the ITS (Internal Transcribed Spacer)-5.8S rDNA region sequence [10]. The strain was deposited in the Green Pesticide Development Laboratory, Qingdao Agricultural University. *F. oxysporum* strains, as well as weed seeds of *E. crusgalli*, *D. sanguinalis*, *P. oleracea,* and *D. sophia,* were provided by the College of Plant Disease, Qingdao Agricultural University, while carbendazim-resistant strains of *B. cinerea* were isolated and identified by the Green Pesticide Development Laboratory.

### 3.3. Fermentation, Extraction, and Isolation

The fungus *A. iridiaustralis* was transferred to PDA medium and cultured at 28 °C for 7 days. Then pieces of fresh mycelia were inoculated and statically fermented at 28 °C for 30 days on the solid rice medium, which was conducted in 40 × 1 L conical flasks containing rice (100 g/flask), peptone (0.6 g/flask), and natural seawater (100 mL/flask).

The fungal culture was exhaustively extracted using ethyl acetate (EtOAc) to obtain a crude extract (12.6 g), which was fractionated via silica gel vacuum liquid chromatography with the eluting gradient of petroleum ether/EtOAc (40:1, 20:1, 10:1, 5:1, and 1:1) and then dichloromethane (CH_2_Cl_2_)/methanol (MeOH) (20:1, 10:1, 5:1, and 1:1) to yield nine fractions [Fractions (Frs.) 1–9].

Antagonistic Fr. 5 was purified via CC over RP-C18 eluting with a MeOH−H_2_O gradient (from 1:9 to 1:0) to obtain six subfractions (Fr.5-1 to 5-6). Fr.5-2 was isolated via CC over Sephadex LH-20 (MeOH) to yield two subfractions; one was purified using semi-pHPLC (35% MeOH−H_2_O) to obtain compounds **5** (8.2 mg, t*_R_* 11.2 min), **6** (12.3 mg, t*_R_* 14.5 min), and **7** (9.1 mg, t*_R_* 17.9 min), while the other used semi-pHPLC (40% MeOH−H_2_O) to obtain compound **8** (4.4 mg, t*_R_* 13.7 min). Fr.5-3 was first separated via CC over Sephadex LH-20 (MeOH) and then purified using semi-pHPLC (53% MeOH−H_2_O) to obtain compounds **9** (21.7 mg, t*_R_* 16.3 min) and **4** (13.8 mg, t*_R_* 19.7 min). Fr.5-4 was first isolated via semi-pHPLC (70% MeOH−H_2_O) and then via CC over Sephadex LH-20 (acetone) to obtain compounds **1** (6.6 mg), **2** (9.2 mg), and **3** (6.8 mg).

*16-methoxy solanapyrone B (**1**)*: White powder. [*α*${]}_{D}^{24}$ = –42.4, *c* 1.15, CHCl_3_; UV (CH_3_OH) *λ*_max_ (log *ε*) 205 (2.31), 302 (1.15) nm; ECD (CH_3_OH) *λ*_max_ ([θ]) 298 (–30.64), 207 (+59.15) nm; ^1^H and ^13^C NMR data, see Table 1; HRESIMS *m/z* 319.19006 [M + H]^+^ (calcd for C_19_H_27_O_4_, 319.19039; Mass error: –3.26 ppm).

*Solanapyrone S (**3**)*: White powder. [*α*${]}_{D}^{24}$ = –53.7, *c* 1.22, CHCl_3_; UV (CH_3_OH) *λ*_max_ (log *ε*) 206 (2.29), 304 (1.08) nm; ECD (CH_3_OH) *λ*_max_ ([θ]) 298 (–21.92), 206 (+88.03) nm; ^1^H and ^13^C NMR data, see Table 1; HRESIMS *m/z* 399.21359 [M + Na]^+^ (calcd for NaC_22_H_32_O_5_, 399.21420; Mass error: –6.05 ppm).

*Alternanone A (**5**)*: White powder. [*α*${]}_{D}^{24}$ = +11.4, *c* 0.94, CH_3_OH; UV (CH_3_OH) *λ*_max_ (log *ε*) 206 (4.37), 238 (3.81), 255 (4.02), 298 (3.06) nm; ^1^H and ^13^C NMR data, see Table 2; HRESIMS *m/z* 299.05847 [M + H]^+^ (calcd for C_13_H_15_O_6_S, 299.05839; Mass error: 0.85 ppm), 219.06537 [M—SO_2_CH_3_]^+^.

*Alternanone B (**6**)*: White powder. [*α*${]}_{D}^{24}$ = +9.8, *c* 0.93, CH_3_OH; UV (CH_3_OH) *λ*_max_ (log *ε*) 205 (4.47), 235 (3.72), 254 (4.06), 296 (3.11) nm; ^1^H and ^13^C NMR data, see Table 2; HRESIMS *m/z* 205.08658 [M + H]^+^ (calcd for C_12_H_13_O_3_, 205.08592; Mass error: 6.59 ppm).

*Alternanone C (**8**)*: White powder. [*α*${]}_{D}^{24}$ = +8.4, *c* 1.07, CH_3_OH; UV (CH_3_OH) *λ*_max_ (log *ε*) 208 (2.31), 242 (2.04), 328 (1.03) nm; ECD (CH_3_OH) *λ*_max_ ([θ]) 330 (–2.45), 240 (+17.12), 212 (–3.93) nm; ^1^H and ^13^C NMR data, see Table 2; HRESIMS *m/z* 227.16408 [M + H]^+^ (calcd for C_13_H_23_O_3_, 227.16417; Mass error: –0.91 ppm).

### 3.4. Calculations of ECD and ^13^C NMR Data

Conformational searches were carried out by means of the Merck Molecular Force Field (MMFF) using Spartan’s 10 software. The conformers with a Boltzmann population over 1% were chosen for ECD and ^13^C NMR data calculations. The optimized geometries of predominant conformers (weighting factors) for compounds **1**, **3,** and **8** at the B3LYP/6-311++g(d, p) level above 1% population were shown in Figures S36–S38, respectively. Further calculations of their ECD and ^13^C NMR data were performed as described previously [10,17,21].

### 3.5. Herbicidal and Antifungal Evaluations

Herbicidal bioassays of compounds **1**–**9** against *E. crusgalli* were performed using the grinded plant tissue powders mixed with agar method as described previously [9,10]. Briefly, weed seeds were pretreated with sodium hypochlorite (0.2%) for 15 min and then soaked with flowing water for 4–6 h. Wet seeds were germinated for 12 h on the moist filter paper under 28 °C in a dark condition. Isolated compounds were dissolved with methanol to obtain sample solutions of different concentrations. 1 mL sample solution and 99 mL water (containing 0.5 g agar) were mixed to yield the agar solution (1% methanol), which was further divided into three beakers. Subsequently, germinated seeds with the same radicle lengths were planted into beakers and then cultivated in the artificial climate box under a 28 °C light-avoidance condition. After 3 days, the stem and root lengths of weed seedlings were measured and compared to the untreated control. The inhibition rate was calculated using the formula as follows:

Inhibition rate (%) = [(*L*_control_ − *L*_treatment_)/*L*_control_] × 100

Due to the significant herbicidal potential of **9**, its herbicidal spectrum was further evaluated using malignant weeds *D. sanguinalis*, *P. oleracea*, and *D. Sophia*, which were widely distributed in North China, resulting in serious economic losses.

Antifungal bioassays of isolated metabolites (**1**–**9**) against two groups of representative soil-borne phytopathogens, including carbendazim-resistant isolates of *B. cinerea* from grape (BCG) and strawberry (BCS), as well as *F. oxysporum* strains of *F. oxysporum* f. sp. *cucumerinum* (FOC) and *F. oxysporum* f. sp. *Lycopersici* (FOL), were performed using the broth microdilution method in 96-well plates [17,18]. Briefly, isolated compounds, dissolving in 50% DMSO aqueous solution, were added 5 μL per well to 95 μL Potato Dextrose Broth (PDB). 96-well plates with *B. cinerea* and *F. oxysporum* strains were cultivated at 25 °C and 28 °C for three days, respectively.

## 4. Conclusions

An investigation of agricultural bioactive metabolites from the marine-derived fungus *A. iridiaustralis* obtained nine metabolites (**1**–**9**), including five novel ones (**1**, **3**, **5**–**6**, and **8**). Their structures and absolute configurations were elucidated by extensive spectroscopic analyses as well as comparisons between measured and calculated ECD and ^13^C NMR data. Compounds **1**–**4**, **6,** and **9** showed herbicidal potential against the radicle growth of *E. crusgalli* seedlings. Especially **9** exhibited inhibition rates over 90% at concentrations of 20 and 40 μg/mL, even better than the chemical herbicide acetochlor. Furthermore, **9** also performed a wide herbicidal spectrum against malignant weeds *D. sanguinalis*, *P. oleracea*, and *D. Sophia*. Compounds **5**–**8** showed antifungal activities against carbendazim-resistant strains of *B. cinerea* that were better than those of carbendazim. Although the antifungal activity of **6** was weaker than that of **8**, its integrated agricultural potential against both weeds and soil-borne pathogens indicated its application in the development of bio-pesticides. Overall, the marine-derived fungus *A. iridiaustralis*, which produces herbicidal and antifungal metabolites **1**–**9**, showed the potential for use as a microbial pesticide to control both weed and soil-borne pathogens.

## Figures and Tables

**Table 1:** ^1^H (500 MHz) and ^13^C (125 MHz) NMR data of **1** and **3** (CD_3_OD, *δ*: ppm).

	Compound 1		Compound 3	
No.	*δ*_C_ (type)	*δ*_H_ (Mult., *J* in Hz)	*δ*_C_ (type)	*δ*_H_ (Mult., *J* in Hz)
1	47.7, CH	2.63, t (10.9)	47.7, CH	2.80, dd (11.7, 10.0)
2	36.5, CH	2.55, m	36.5, CH	2.71, m
3	131.6, CH	5.47, dd (10.5, 2.0)	131.6, CH	5.64, dd (10.0, 1.7)
4	132.7, CH	5.69, dd (10.5, 2.5)	132.7, CH	5.85, dd (10.0, 2.6)
5	38.5, CH	2.16, m	38.5, CH	2.32, m
6	30.9, CH_2_	1.71, m; 1.25, m	30.9, CH_2_	1.87, m; 1.41, m
7	29.5, CH_2_	1.38, m	29.5, CH_2_	1.56, m
8	27.3, CH_2_	1.73, m; 1.22, m	27.3, CH_2_	1.91, m; 1.36, m
9	21.9, CH_2_	1.45, m	21.9, CH_2_	1.62, m
10	37.6, CH	2.24, m	37.6, CH	2.40, m
11	170.8, C		170.6, C	
12	98.6, CH	6.67, s	98.7, CH	6.84, s
13	171.5, C		171.1, C	
13-OCH_3_	57.9, CH_3_	3.99, s	57.9, CH_3_	4.15, s
14	101.4, C		102.1, C	
15	167.5, CO		167.8, CO	
16	64.1, CH_2_	4.31, s	61.3, CH_2_	4.68, d (10.4); 4.52, d (10.4)
16-OCH_3_	58.3, CH_3_	3.33, s		
17	20.5, CH_3_	0.96, d (7.0)	20.5, CH_3_	1.12, d (7.0)
1′			15.6, CH_3_	1.29, d (6.3)
2′			81.0, CH	3.48, ov
3′			71.6, CH	3.79, m
4′			18.4, CH_3_	1.26, d (6.4)

ov: overlapped ^1^H NMR signals.

**Table 2:** ^1^H (500 MHz) and ^13^C (125 MHz) NMR data of **5**–**6** and **8** (*δ*: ppm).

	Compound 5		Compound 6			Compound 8	
No.	*δ*_C_	*δ*_H_	*δ*_C_	*δ*_H_	No.	*δ*_C_	*δ*_H_
1	167.3, C		165.7, C		1	203.0, CO	
2	109.3, CH	6.10, s	111.5, CH	5.97, s	2	126.1, CH	5.88, s
3	182.5, C		182.4, C		3	161.0, C	
4	105.2, C		115.1, C		4	32.0, CH_2_	2.63, dd (18.3, 3.7) 2.53, dd (18.3, 6.0)
5	159.9, C		140.8, C		5	73.1, CH	4.02, m
6	101.2, C		124.7, C		6	49.9, C	
7	164.0, C		162.3, C		7	18.5, CH_3_	1.09, s
8	90.6, CH	6.45, s	100.7, CH	6.65, s	8	23.4, CH_2_	1.70, q (7.5)
9	158.6, C		159.4, C		9	7.7, CH_3_	0.86, t (7.5)
10	20.7, CH_3_	2.39, s	19.7, CH_3_	2.30, s	1′	15.5, CH_3_	1.08, ov
11	49.4, CH_2_	4.45, s	17.6, CH_3_	2.75, s	2′	49.6, CH	2.30, m
12			11.6, CH_3_	2.17, s	3′	70.2, CH	3.80, dt (12.9, 6.0)
5-OH		13.35, s			4′	21.4, CH_3_	1.25, d (6.0)
*S*CH_3_	41.5	2.91, s					
7-OCH_3_	56.6	3.95, s					

Compounds **5** and **8** were determined using CDCl_3_, while **6** using CD_3_OD (^1^H of 500 MHz and ^13^C of 125 MHz), respectively; ov: overlapped ^1^H NMR signals.

**Table 3:** Herbicidal (Inhibition rates: %) and antifungal (MIC: μg/mL) potentials of the isolated metabolites **1**–**9**.

No.	Herbicidal Activities			Antifungal Activities			
	40 μg/mL	20	10	BCG	BCS	FOC	FOL
**1**	57.1 ± 3.1c	45.4 ± 2.9d	<20	—	—	—	—
**2**	61.3 ± 2.4c	50.4 ± 1.2d	23.7 ± 2.6c	—	—	—	—
**3**	43.1 ± 1.7d	28.9 ± 3.0f	n.d.	—	—	—	—
**4**	50.4 ± 2.3cd	36.2 ± 3.5e	n.d.	—	—	—	—
**5**	—	n.d.	n.d.	64	64	—	—
**6**	72.6 ± 1.9b	60.3 ± 2.4c	30.1 ± 2.2b	64	64	128	256
**7**	—	n.d.	n.d.	128	128	256	256
**8**	—	n.d.	n.d.	32	32	128	256
**9**	98.3 ± 0.3a	90.2 ± 1.5a	67.3 ± 2.7a	—	—	—	—
CK	91.7 ± 3.4a	80.4 ± 2.1b	74.2 ± 1.5a	256	256	8	8

CK of herbicidal and antifungal bioassays were commonly used chemical pesticides acetochlor and carbendazim, respectively; “—”: no activity; n.d.: not detected. Different lowercase letters in a column indicated the means were significantly different at *p* < 0.05.

## Data Availability

The research data were available in Supporting Information.

## Supplementary Materials

The following supporting information can be downloaded at: https://www.mdpi.com/article/10.3390/jof9070716/s1, 1D and 2D NMR spectra, HRESIMS data of compounds **1**, **3**, **5**–**6** and **8** are available as Supporting Information. The Figures S1–S40 and Table S1 were listed in Supporting Information.

## References

[1] Gianessi L.P. (2013). The increasing importance of herbicides in worldwide crop production. Pest Manag. Sci..

[2] Dayan F.E., Duke S.O. (2014). Natural compounds as next-generation herbicides. Plant Physiol..

[3] Yin C.P., Jin L.P., Sun F.F., Xu X., Shao M.W., Zhang Y.L. (2018). Phytotoxic and antifungal metabolites from *Curvularia crepinii* QTYC-1 isolated from the gut of *Pantala flavescens*. Molecules.

[4] Dean R., Van K.J.A.L., Pretorius Z.A., Hammond-Kosack K.E., Di Pietro A., Spanu P.D., Rudd J.J., Dickman M., Kahmann R., Ellis J. (2012). The top 10 fungal pathogens in molecular plant pathology. Mol. Plant Pathol..

[5] Russell P.E. (2006). The development of commercial disease control. Plant Pathol..

[6] Cantrell C.L., Dayan F.E., Duke S.O. (2012). Natural products as sources for new pesticides. J. Nat. Prod..

[7] Song X.Q., Yang N., Su Y.H., Lu X.Y., Liu J., Liu Y., Zhang Z.H., Tang Z.H. (2022). *Suaeda glauca* and *Suaeda salsa* employ different adaptive strategies to cope with saline-alkali environments. Agronomy.

[8] Xiao L., Niu H.J., Qu T.L., Zhang X.F., Du F.Y. (2021). *Streptomyces* sp. FX13 inhibits fungicide-resistant *Botrytis cinerea* in vitro and in vivo by producing oligomycin A. Pestic. Biochem. Phys..

[9] Wang Z.F., Zhang W., Xiao L., Zhou Y.M., Du F.Y. (2020). Characterization and bioactive potentials of secondary metabolites from *Fusarium chlamydosporum*. Nat. Prod. Res..

[10] Wang Z.F., Sun Z.C., Xiao L., Zhou Y.M., Du F.Y. (2019). Herbicidal polyketides and diketopiperazine derivatives from *Penicillium viridicatum*. J. Agric. Food Chem..

[11] Akhter N., Pan C., Liu Y., Shi Y.T., Wu B. (2019). Isolation and structure determination of a new indene derivative from endophytic fungus *Aspergillus flavipes* Y-62. Nat. Prod. Res..

[12] Niu X.G., Song L.C., Han M., Xiao Y.N. (2012). Diversity of endophytic fungi of *Suaeda heteroptera* Kitag. Micro. Chin..

[13] Zhao S.Q., Li J., Liu J.P., Xiao S.Y.J., Yang S.M., Mei J.H., Ren M.Y., Wu S.Z., Zhang H.Y., Yang X.L. (2023). Secondary metabolites of *Alternaria*: A comprehensive review of chemical diversity and pharmacological properties. Front. Microbiol..

[14] Deshmukh S.K., Dufosse L., Chhipa H., Saxena S., Mahajan G.B., Gupta M.K. (2022). Fungal endophytes: A potential source of antibacterial compounds. J. Fungi.

[15] Kong K., Huang Z.D., Shi S.P., Pan W.D., Zhang Y.L. (2023). Diversity, antibacterial and phytotoxic activities of culturable endophytic fungi from *Pinellia pedatisecta* and *Pinellia ternate*. BMC Microbiol..

[16] Zhao S.S., Wang B., Tian K.L., Ji W.X., Zhang T.Y., Ping C., Yan W., Ye Y.H. (2021). Novel metabolites from the *Cercis chinensis* derived endophytic fungus *Alternaria alternata* ZHJG5 and their antibacterial activities. Pest Manag. Sci..

[17] Du F.Y., Ju G.L., Xiao L., Zhou Y.M., Wu X. (2020). Sesquiterpenes and cyclodepsipeptides from marine-derived fungus *Trichoderma longibrachiatum* and their antagonistic activities against soil-borne pathogens. Mar. Drugs.

[18] Zhou Y.M., Ju G.L., Xiao L., Zhang X.F., Du F.Y. (2018). Cyclodepsipeptides and sesquiterpenes from marine-derived fungus *Trichothecium roseum* and their biological functions. Mar. Drugs.

[19] Ichihara A., Tazaki H., Sakamura S. (1983). Solanapyrones A, B and C, phytotoxic metabolites from the fungus *Alternaria solani*. Tetrahedron Lett..

[20] Song D., Liang J.J., Pu S.B., Zhang P.P., Peng Y.L., Liu X., Feng T.T., Pu X., Zhou Y., Liu X.W. (2023). Structural elucidation and cytotoxic activity of new monoterpenoid indoles from *Gelsemium elegans*. Molecules.

[21] Du F.Y., Mandi A., Li X.M., Meng L.H., Kurtan T., Wang B.G. (2021). Experimental and computational analysis of the solution and solid-state conformations of hexadepsipeptides from *Beauveria felina*. Chin. J. Chem..

[22] Fujimoto H., Nozawa M., Okuyama E., Ishibashi M. (2002). Five new chromones possessing monoamine oxidase inhibitory activity from an Ascomycete, *Chaetomium quadrangulatum*. Chem. Pharm. Bull..

[23] Li H., Tian J.M., Tang H.Y., Pan S.Y., Zhang A.L., Gao J.M. (2015). Chaetosemins A–E, new chromones isolated from an Ascomycete *Chaetomium seminudum* and their biological activities. RSC Adv..

[24] Oikawa H., Ichihara A., Sakamura S. (1988). Biosynthetic study of betaenone B: Origin of the oxygen atoms and accumulation of a deoxygenated intermediate using P-450 Inhibitor. J. Chem. Soc.-Chem. Commun..

[25] Zhou B., Wang H., Meng B., Wei R., Wang L., An C.F., Chen S.G., Yang C.L., Qiang S. (2019). An evaluation of tenuazonic acid, a potential biobased herbicide in cotton. Pest Manag. Sci..

[26] Yin W.X., Adnan M., Shang Y., Lin Y., Luo C.X. (2018). Sensitivity of *Botrytis cinerea* from nectarine/cherry in China to six fungicides and characterization of resistant isolates. Plant Dis..

