# Supplementary material for: Secondary Metabolites with Herbicidal and Antifungal Activities from Marine-Derived Fungus Alternaria iridiaustralis
Source: J Fungi (Basel). 2023 Jun 29;9(7):716. doi: 10.3390/jof9070716 (PMC10381437; doi:10.3390/jof9070716)
Supplement: Supplementary file 1 [file jof-09-00716-s001.zip › jof-2425881-supplementary.pdf]

Journal of *Fungi*

MDPI

Secondary Metabolites with Herbicidal and Antifungal Activities from Marine-Derived Fungus *Alternaria iridiaustralis*

Jin-qing Fan [1,†], Fang-fang Guo [1,†], Chen Zhao [1], Hong Li [1], Tian-li Qu [2], Lin Xiao [2,*] and Feng-yu Du [2,3*]

Contents

Figure S1. HRESIMS spectrum of compound **1**.	S2
Figure S2. ^{1}H NMR (500 MHz, CD_3OD) spectrum of compound **1**.	S3
Figure S3. ^{13}C NMR spectrum of compound **1**.	S3
Figure S4. DEPT-135 spectrum of compound **1**.	S4
Figure S5. HSQC spectrum of compound **1**.	S4
Figure S6. ^{1}H-^{1}H COSY spectrum of compound **1**.	S5
Figure S7. HMBC spectrum of compound **1**.	S5
Figure S8. NOESY spectrum of compound **1**.	S6
Figure S9. HRESIMS spectrum of compound **3**.	S7
Figure S10. ^{1}H NMR (500 MHz, CD_3OD) spectrum of compound **3**.	S8
Figure S11. ^{13}C NMR spectrum of compound **3**.	S8
Figure S12. DEPT-135 spectrum of compound **3**.	S9
Figure S13. HSQC spectrum of compound **3**.	S9
Figure S14. ^{1}H-^{1}H COSY spectrum of compound **3**.	S10
Figure S15. HMBC spectrum of compound **3**.	S10
Figure S16. NOESY spectrum of compound **3**.	S11
Figure S17. HRESIMS spectrum of compound **5**.	S12
Figure S18. ^{1}H NMR (500 MHz, $CDCl_3$) spectrum of compound **5**.	S13
Figure S19. ^{13}C NMR spectrum of compound **5**.	S13
Figure S20. DEPT-135 spectrum of compound **5**.	S14
Figure S21. HSQC spectrum of compound **5**.	S14
Figure S22. HMBC spectrum of compound **5**.	S15
Figure S23. HRESIMS spectrum of compound **6**.	S16
Figure S24. ^{1}H NMR (500 MHz, CD_3OD) spectrum of compound **6**.	S17
Figure S25. ^{13}C NMR spectrum of compound **6**.	S17
Figure S26. HSQC spectrum of compound **6**.	S18
Figure S27. HMBC spectrum of compound **6**.	S18
Figure S28. HRESIMS spectrum of compound **8**.	S19
Figure S29. ^{1}H NMR (500 MHz, $CDCl_3$) spectrum of compound **8**.	S20
Figure S30. ^{13}C NMR spectrum of compound **8**.	S20
Figure S31. DEPT-135 spectrum of compound **8**.	S21
Figure S32. HSQC spectrum of compound **8**.	S21
Figure S33. ^{1}H-^{1}H COSY spectrum of compound **8**.	S22
Figure S34. HMBC spectrum of compound **8**.	S22
Figure S35. NOESY spectrum of compound **8**.	S23
Figure S36. Optimized geometries of predominant conformers (weighting factors) for compound **1** at the B3LYP/6-311++g(d, p) level above 1% population.	S23
Figure S37. Optimized geometries of predominant conformers (weighting factors) for compound **3** at the B3LYP/6-311++g(d, p) level above 1% population.	S24
Figure S38. Optimized geometries of predominant conformers (weighting factors) for compound **8** at the B3LYP/6-311++g(d, p) level above 1% population.	S24
Figure S39. Regression analyses of experimental versus calculated ^{13}C NMR chemical shifts of compounds **1**, **3** and **8**.	S24

Figure S40. Herbicidal potential of **9** against the radicle growth of *Digitaria sanguinalis* seedlings. S25
Table S1. Herbicidal spectrum of **9** against representative malignant weeds *D. sanguinalis, Portulaca oleracea,* and *Descurainia sophia.* S25

Figure S1. HRESIMS spectrum of compound **1**.

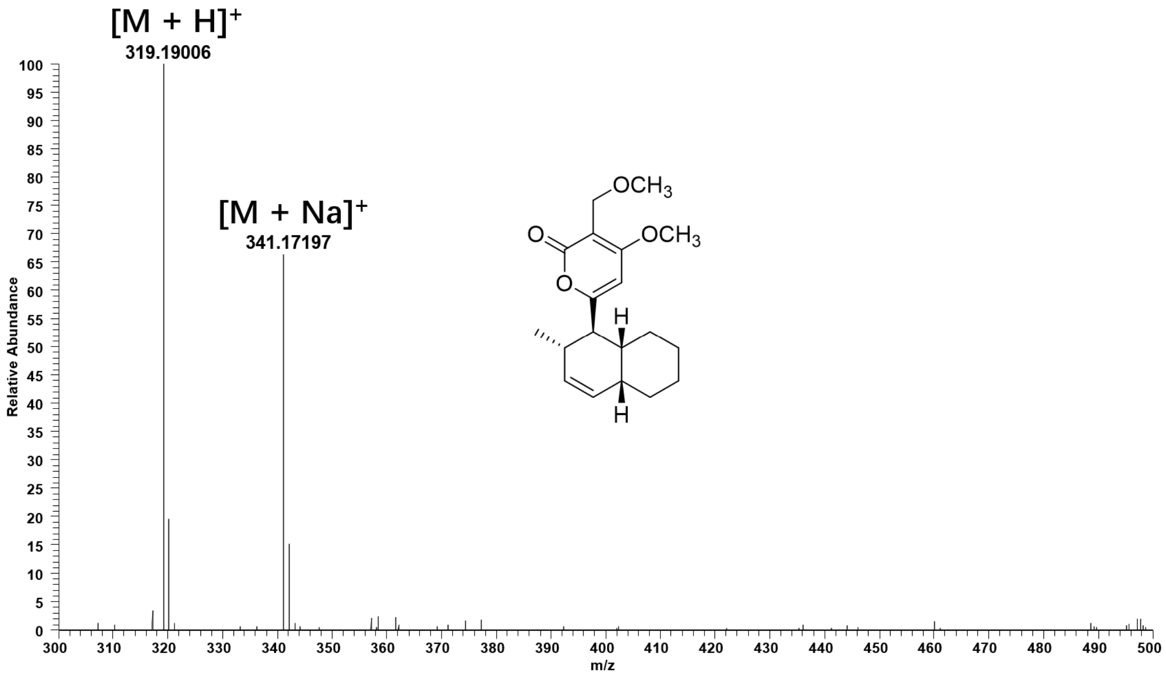

Figure S2. ^{1}H NMR (500 MHz, CD_3OD) spectrum of compound **1**.

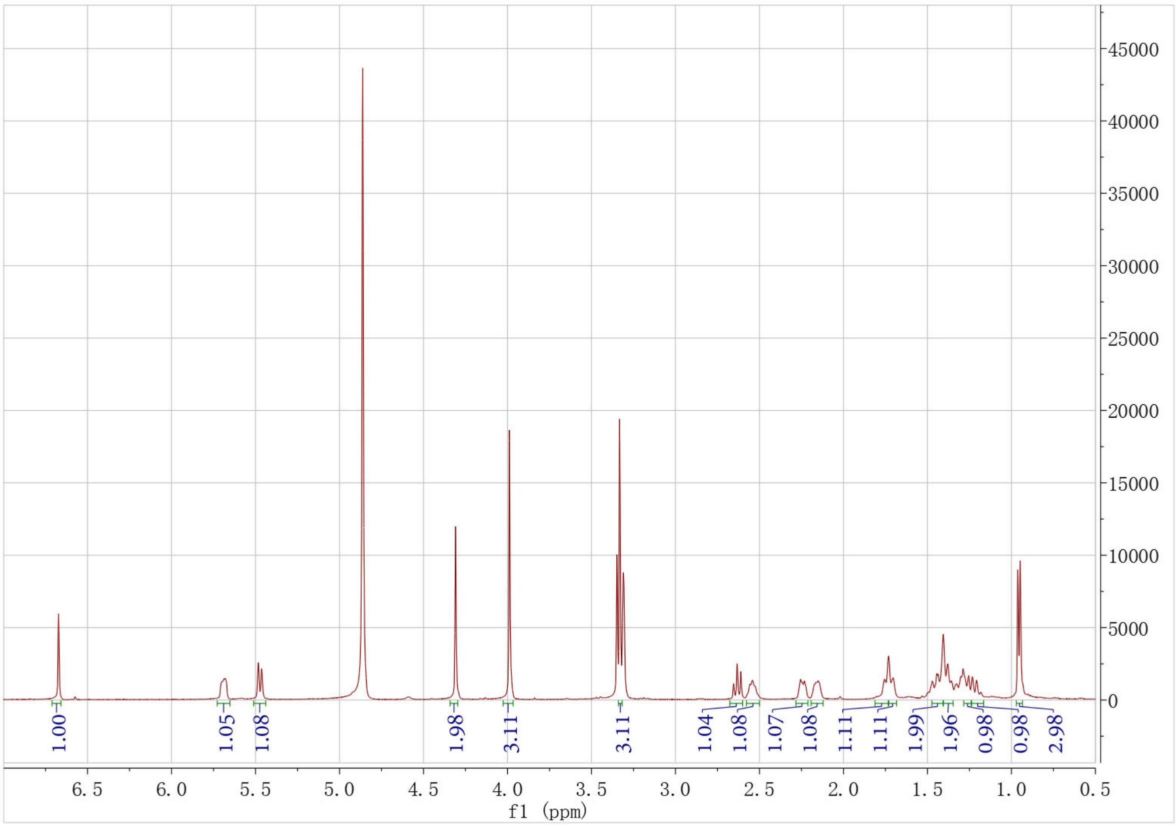

Figure S3. ^{13}C NMR spectrum of compound **1**.

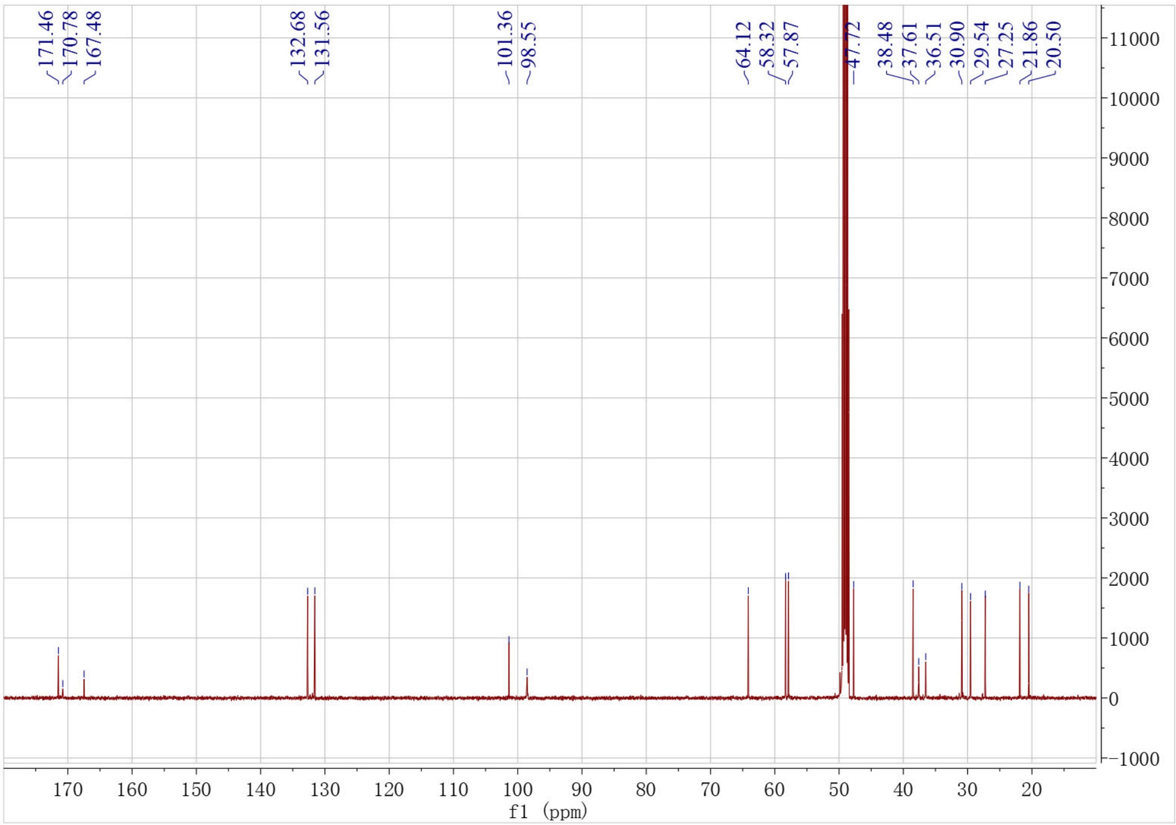

Figure S4. DEPT-135 spectrum of compound **1**.

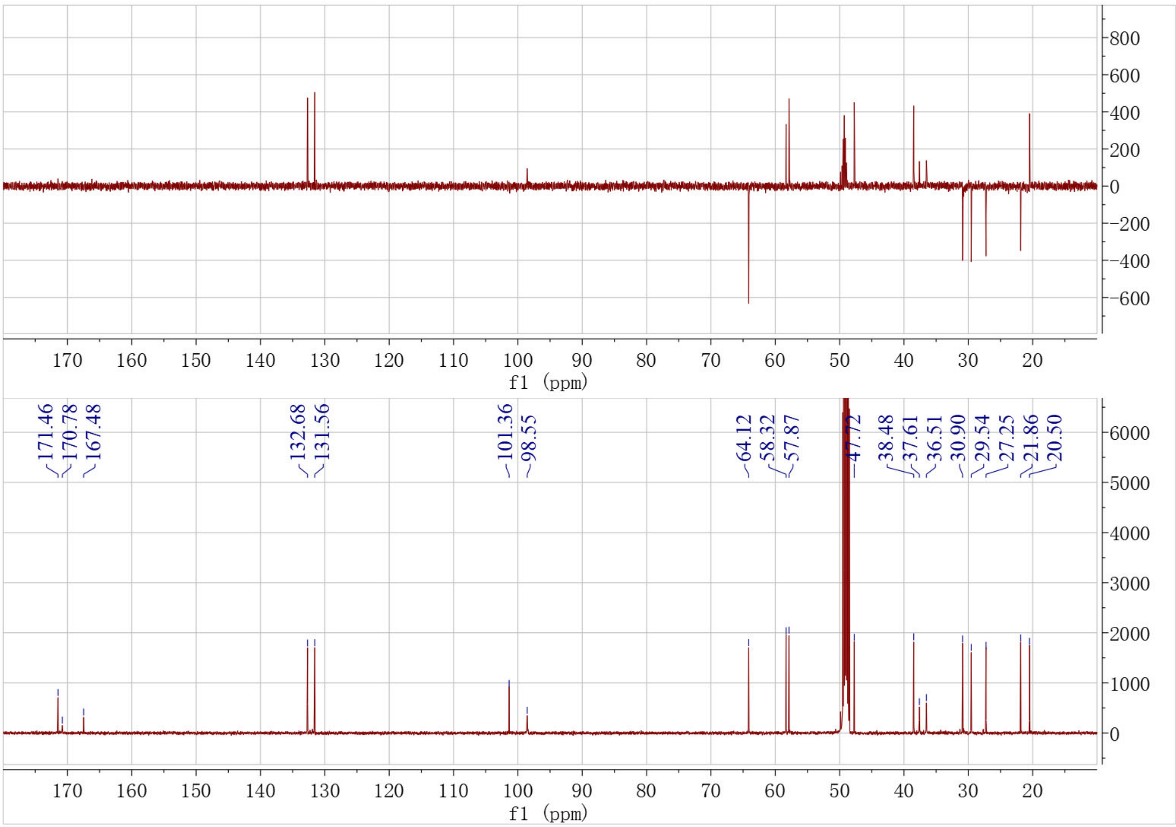

Figure S5. HSQC spectrum of compound **1**.

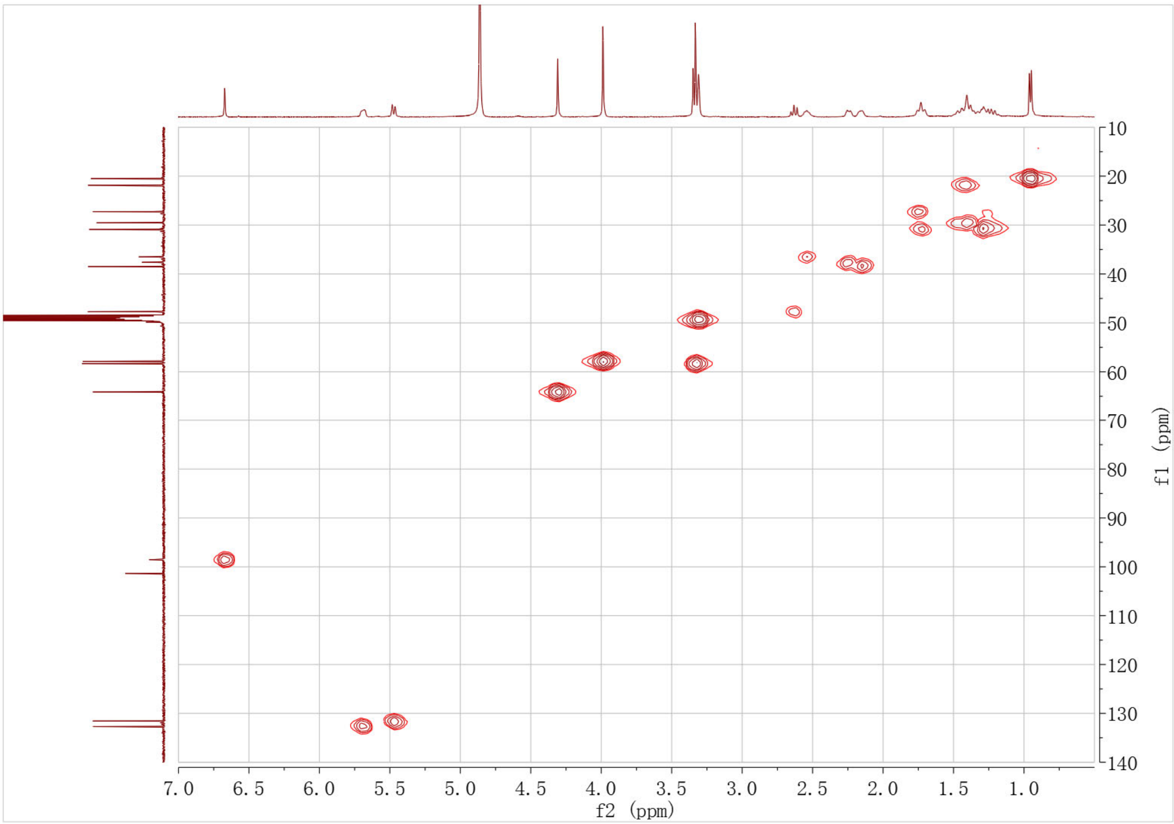

Figure S6. ^{1}H-^{1}H COSY spectrum of compound **1**.

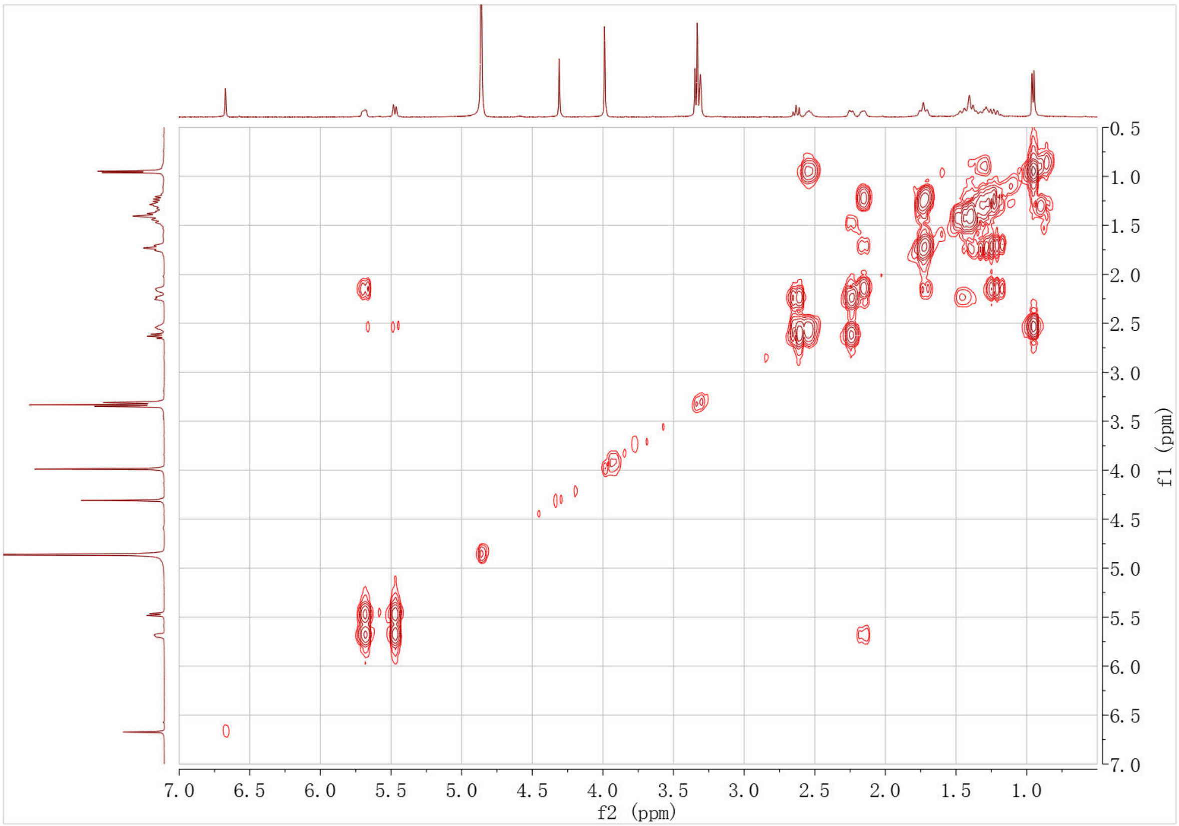

Figure S7. HMBC spectrum of compound **1**.

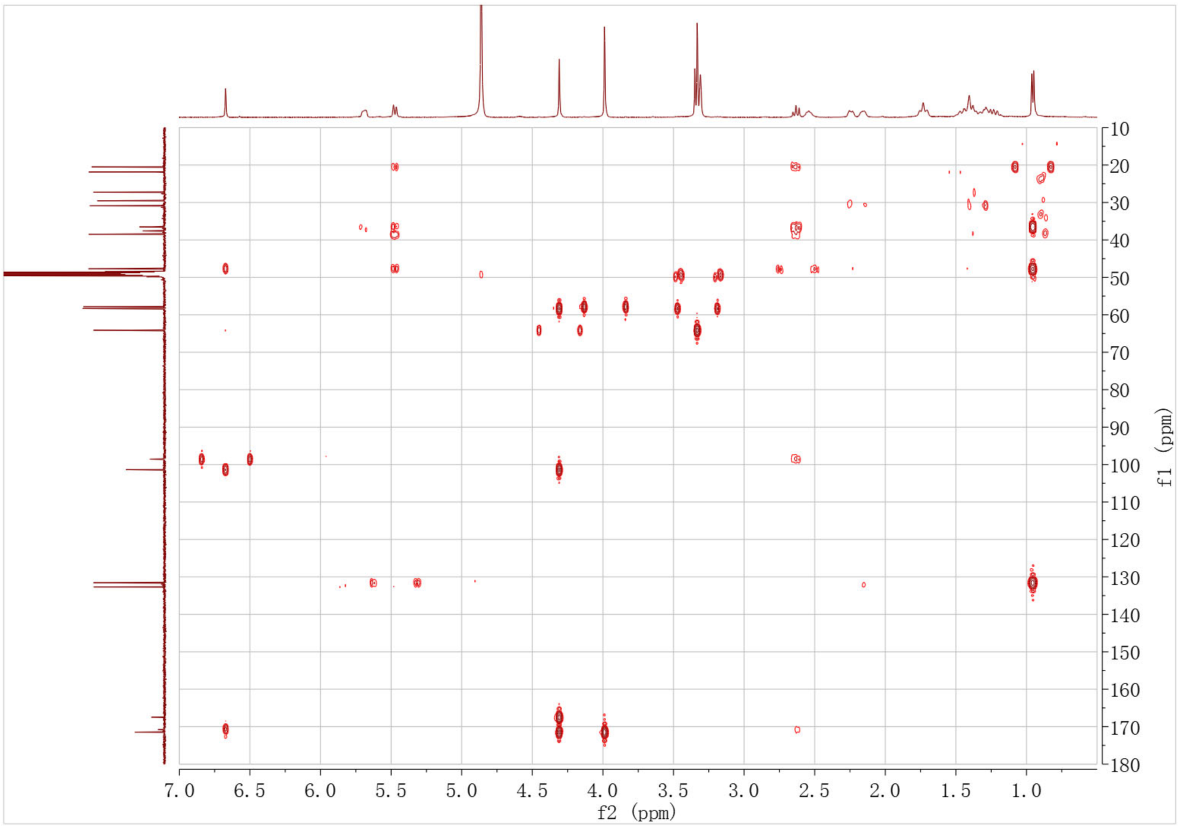

Figure S8. NOESY spectrum of compound **1**.

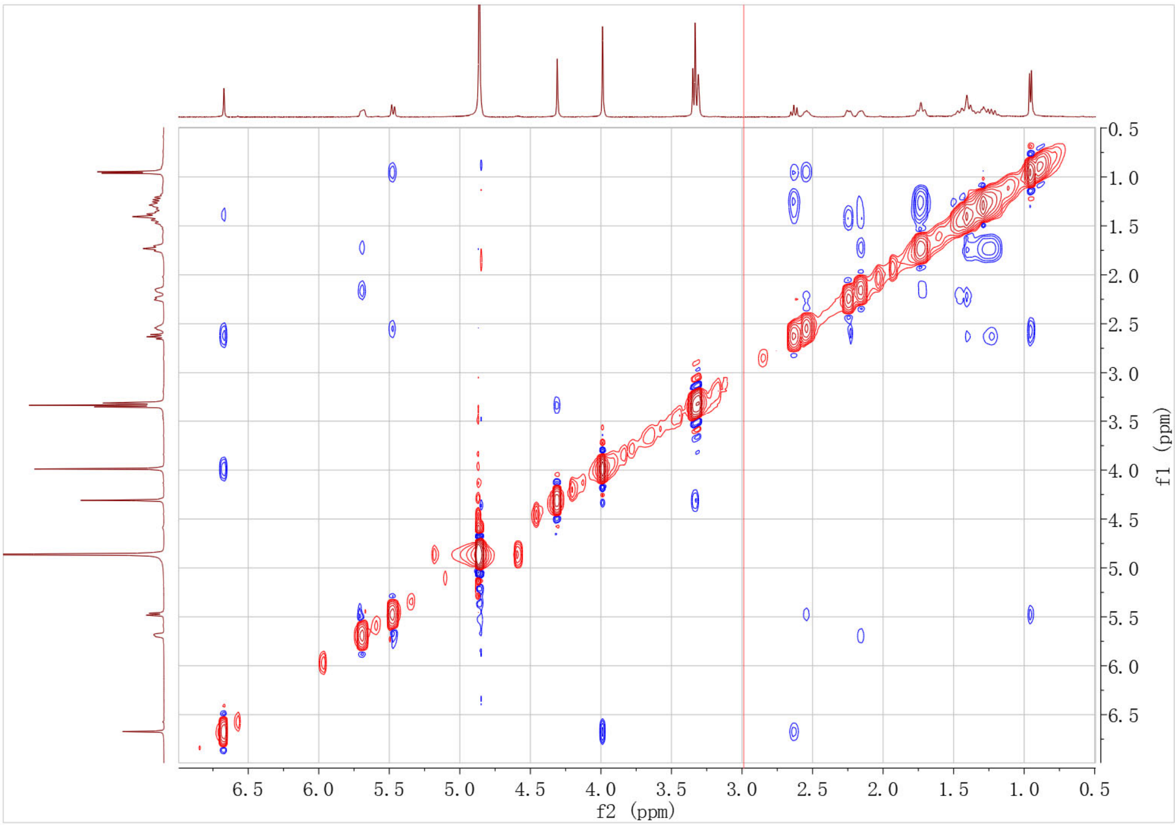

Figure S9. HRESIMS spectrum of compound **3**.

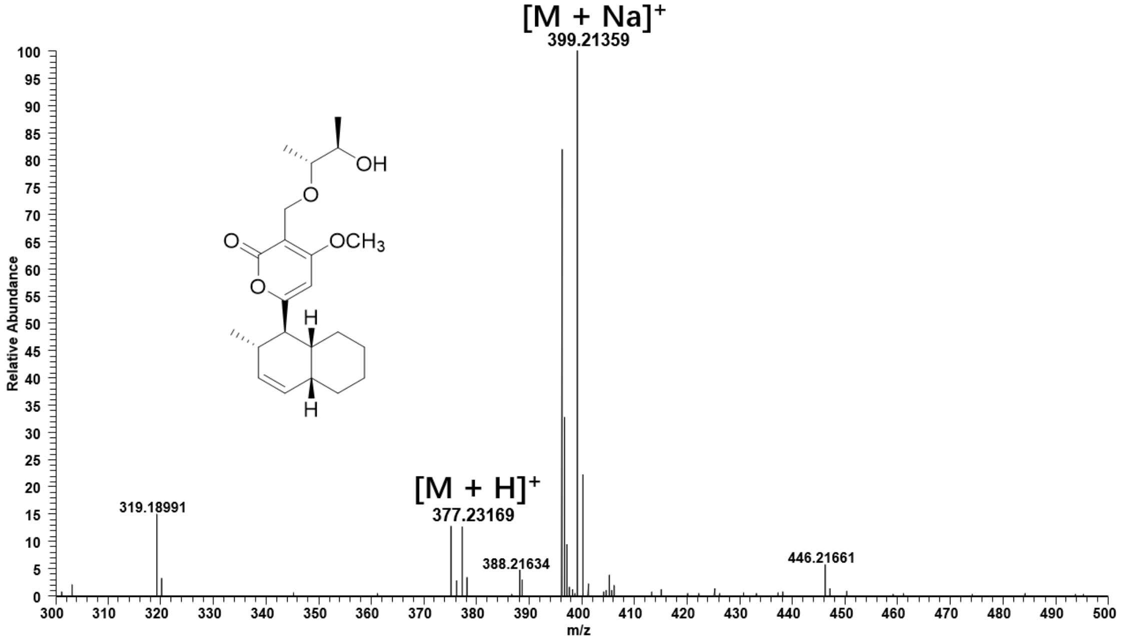

Figure S10. ^{1}H NMR (500 MHz, MeOD) spectrum of compound **3**.

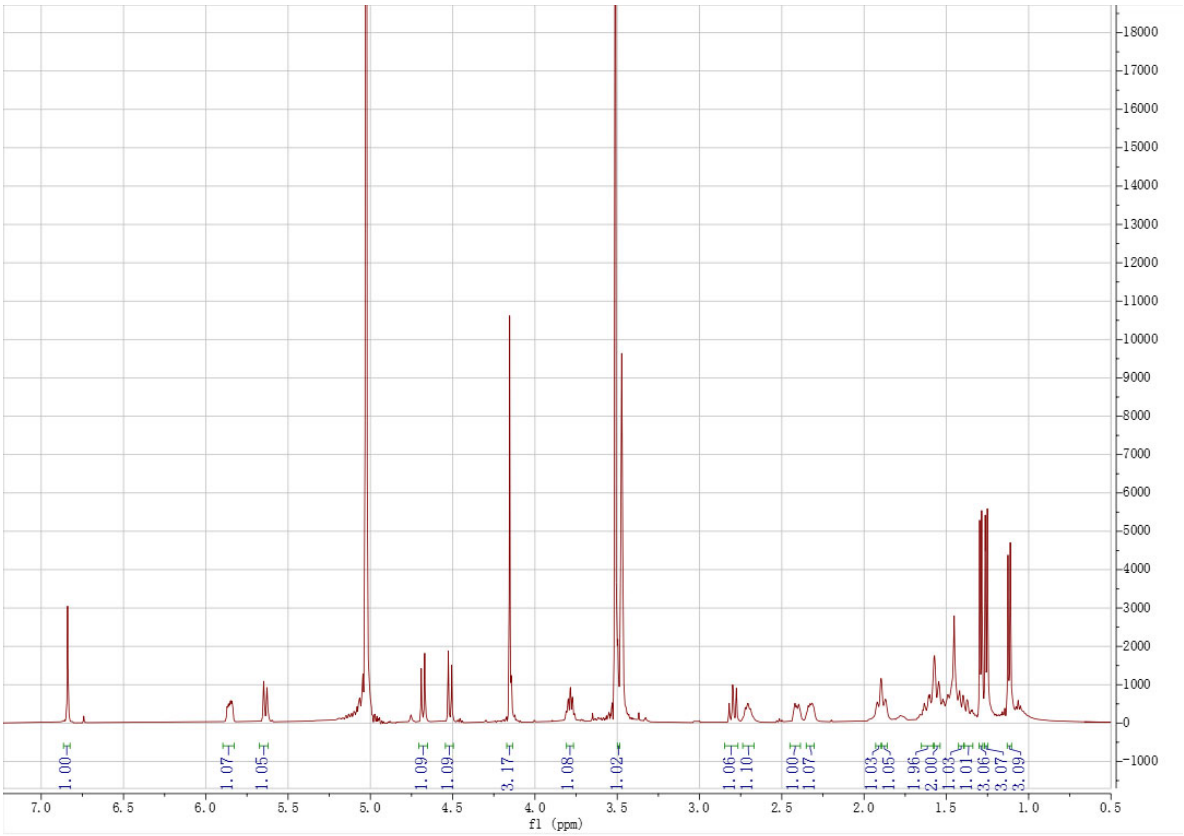

Figure S11. ^{13}C NMR spectrum of compound **3**.

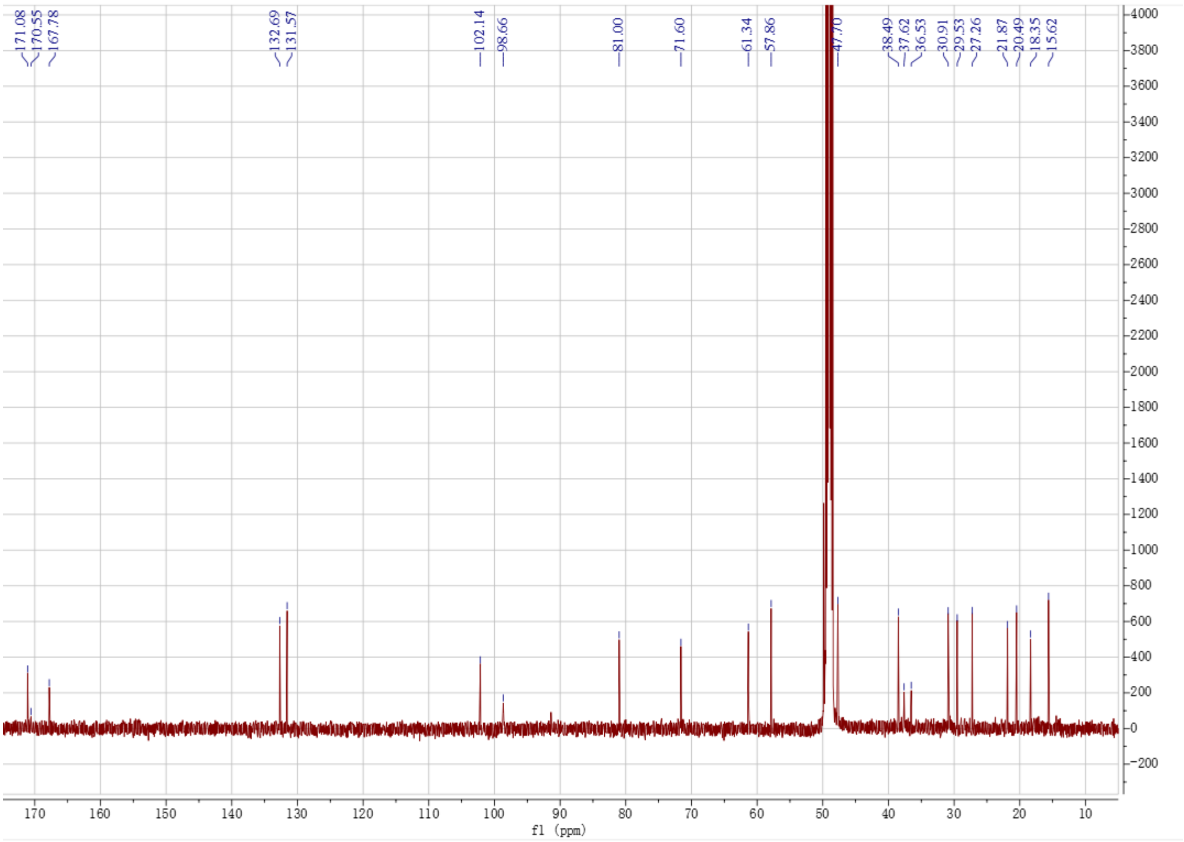

Figure S12. DEPT-135 spectrum of compound **3**.

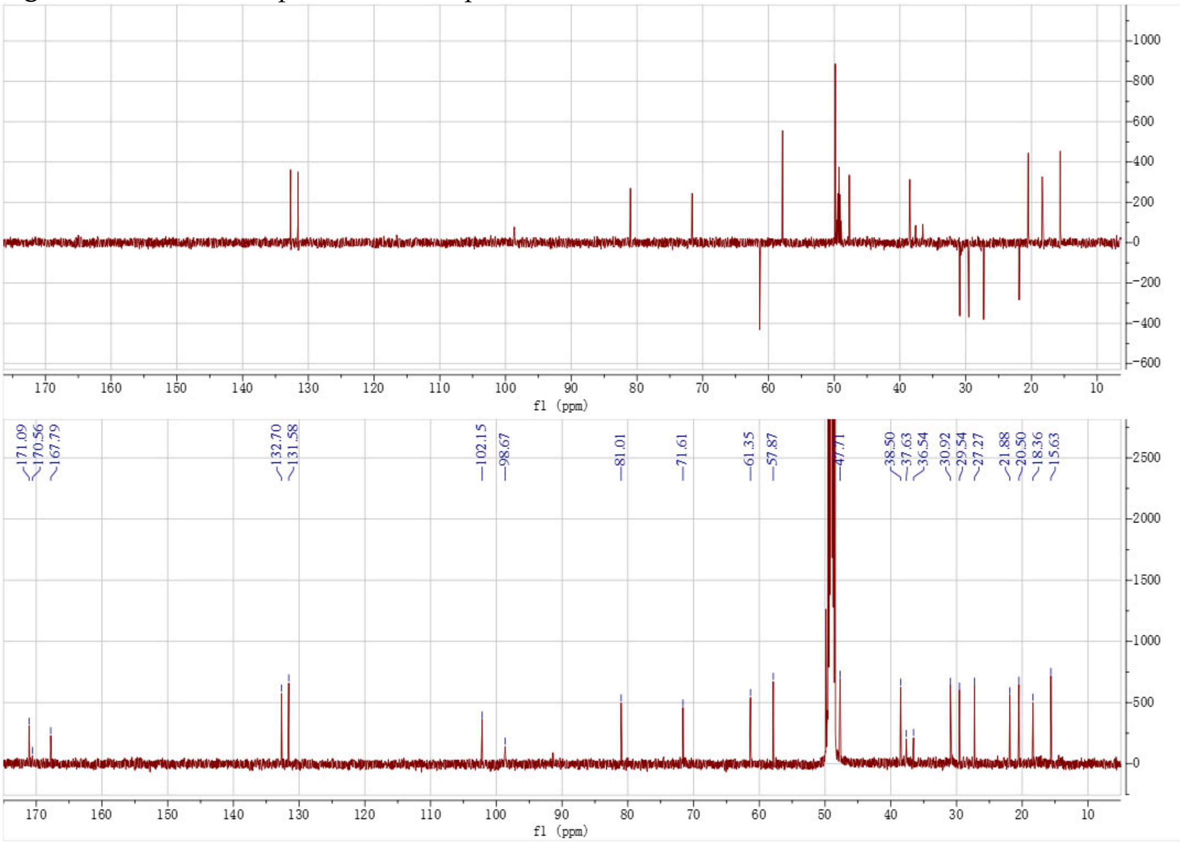

Figure S13. HSQC spectrum of compound **3**.

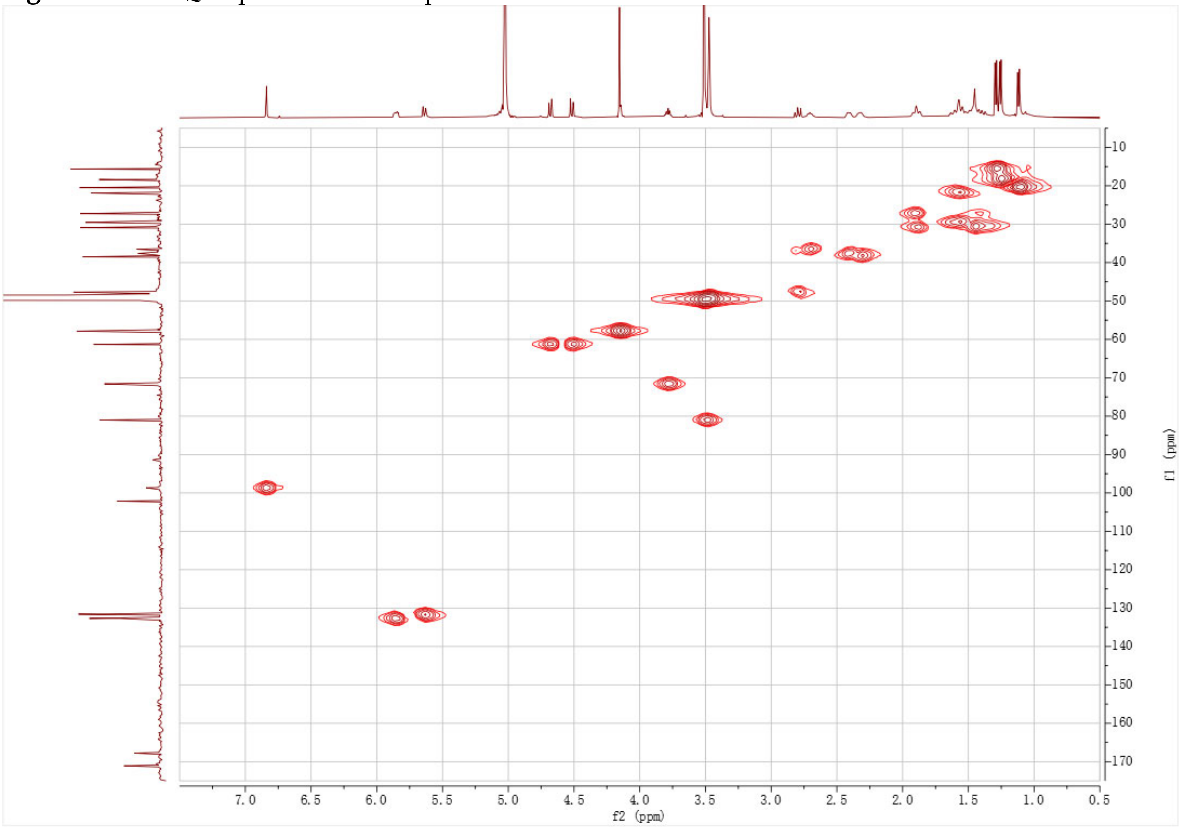

Figure S14. ^{1}H-^{1}H COSY spectrum of compound **3**.

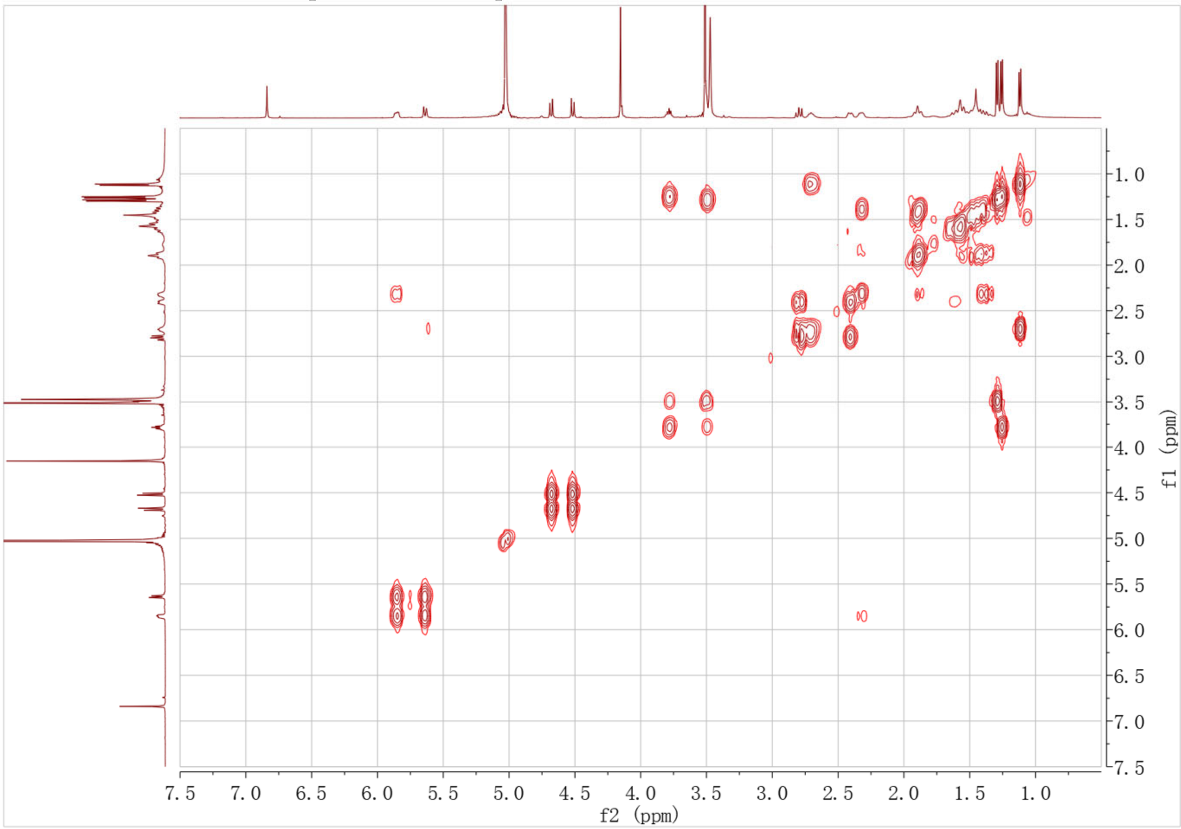

Figure S15. HMBC spectrum of compound **3**.

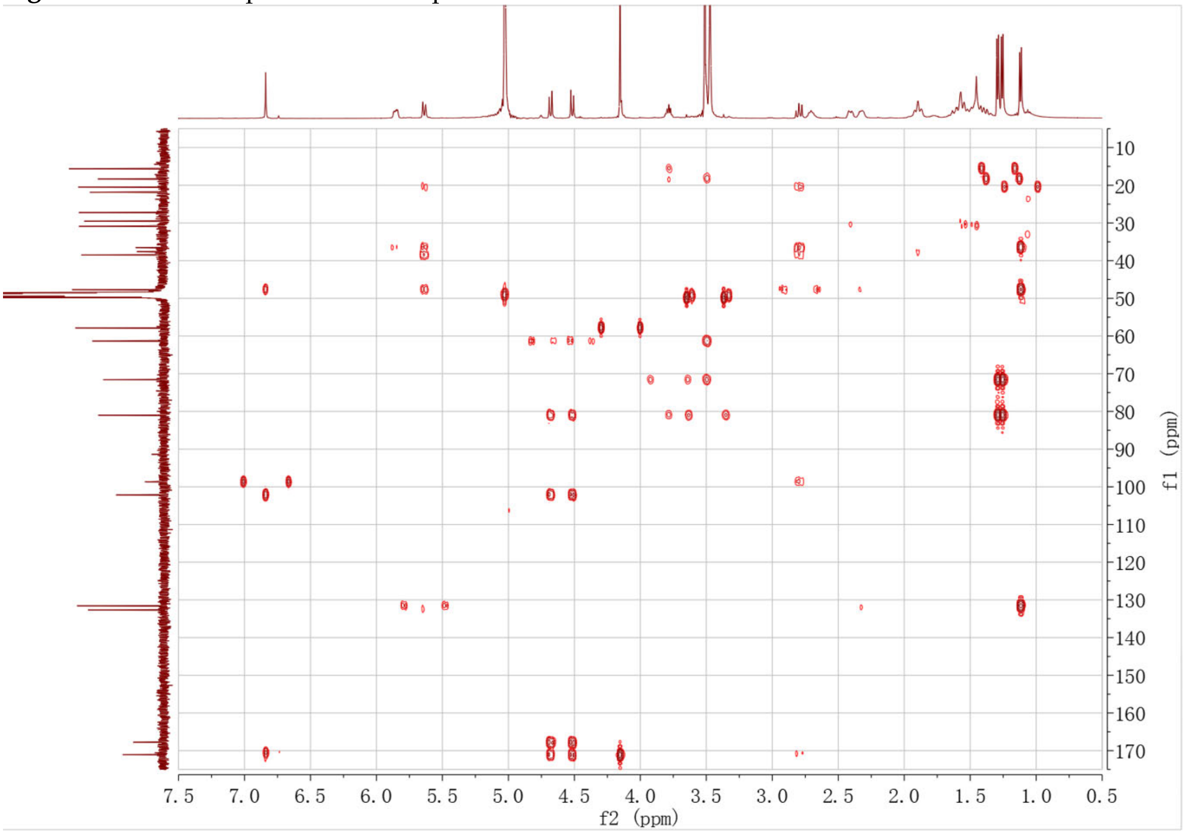

Figure S16. NOESY spectrum of compound **3**.

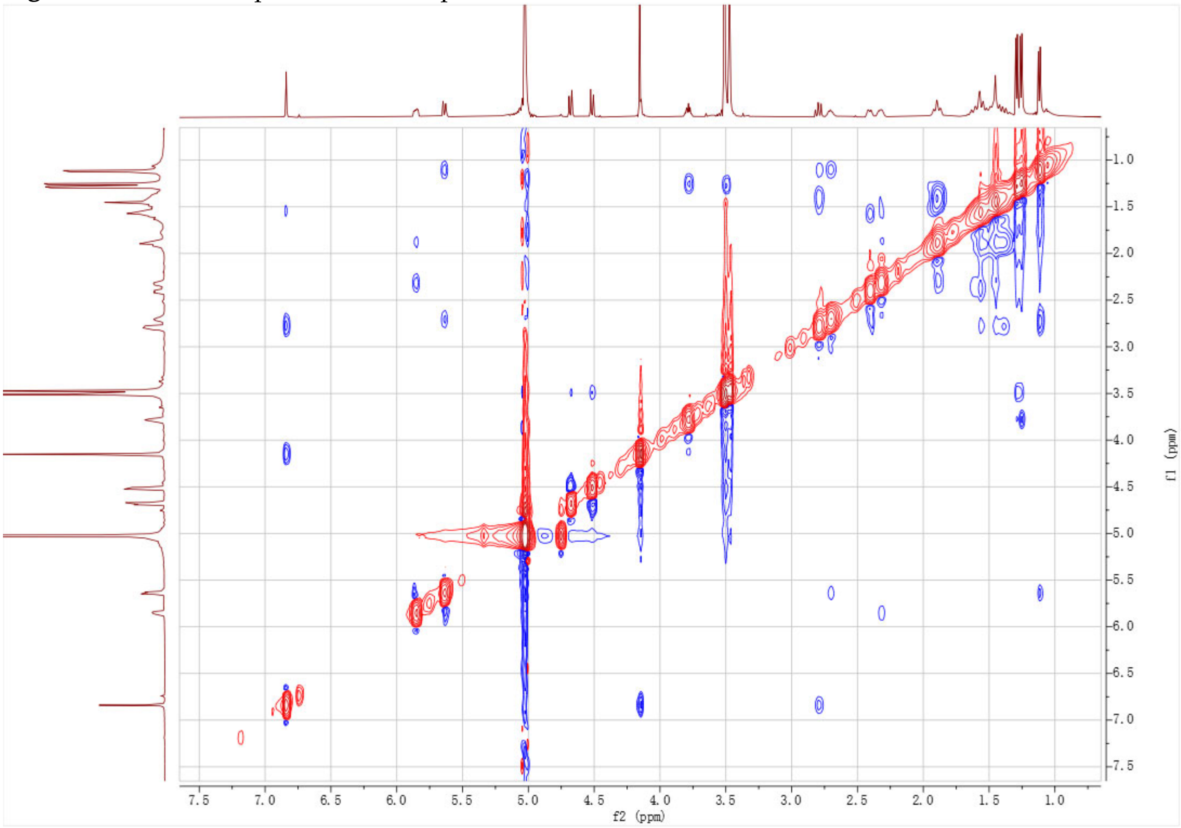

Figure S17. HRESIMS spectrum of compound **5**.

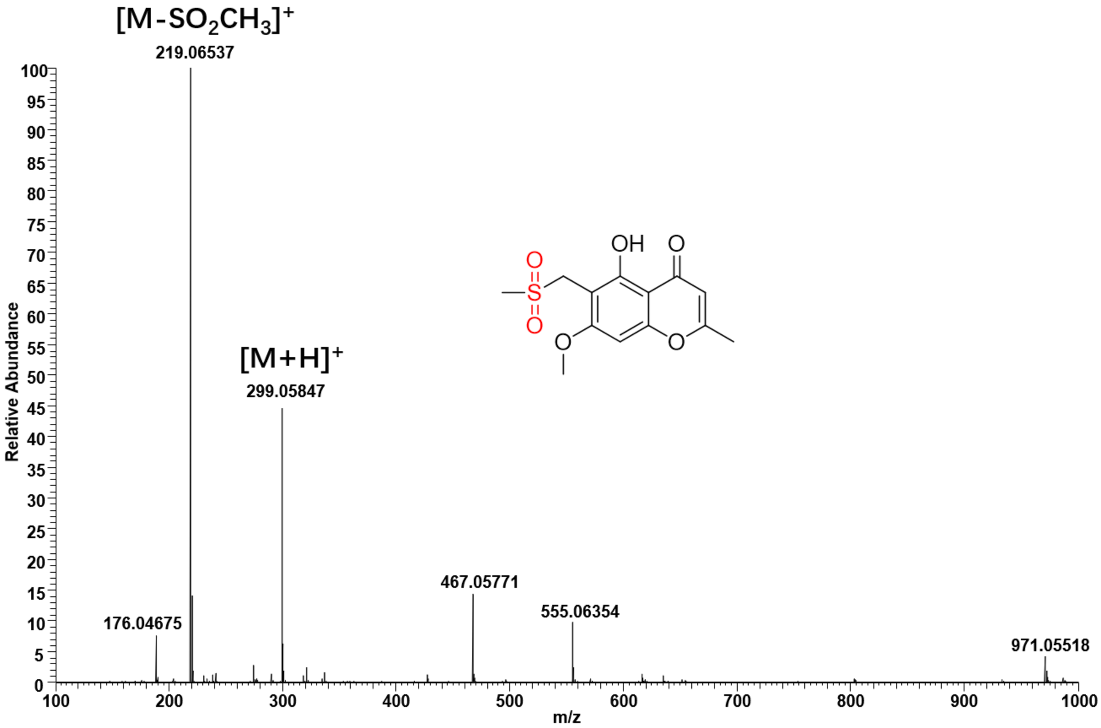

Figure S18. ^{1}H NMR (500 MHz, $CDCl_3$) spectrum of compound **5**.

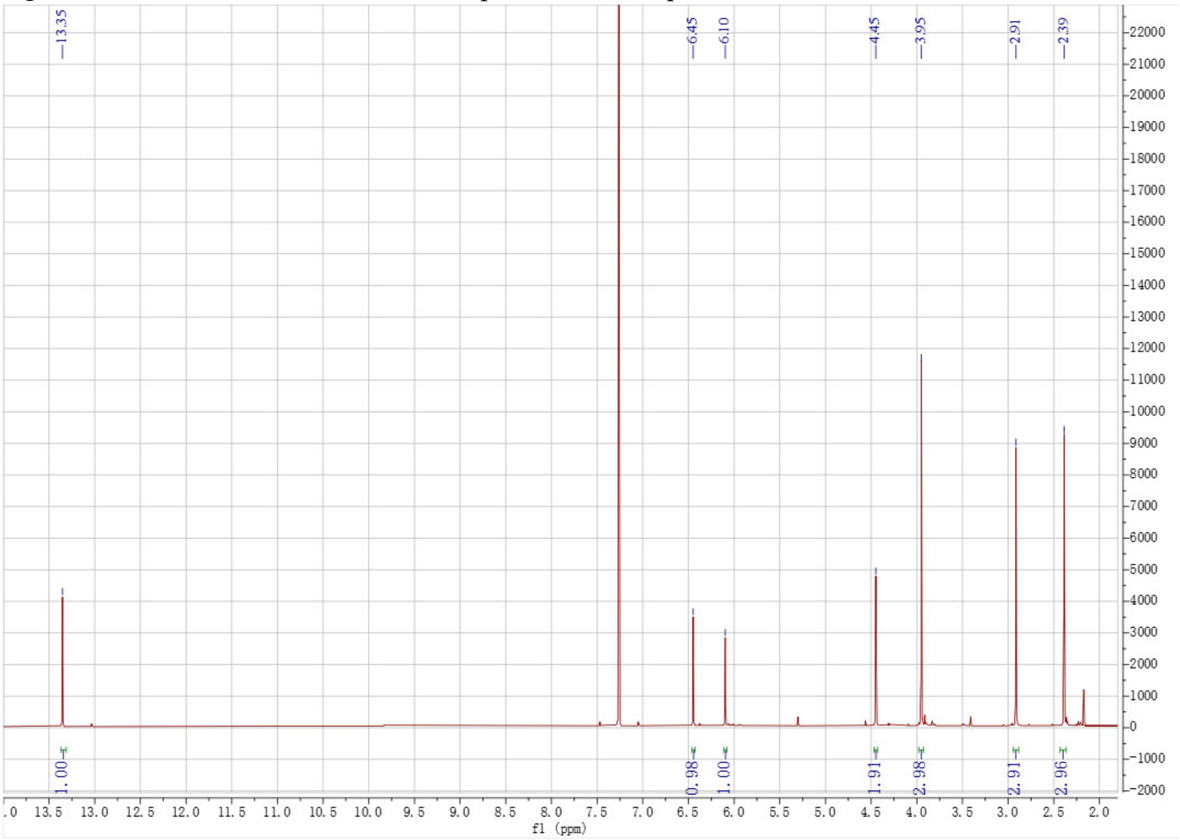

Figure S19. ^{13}C NMR spectrum of compound **5**.

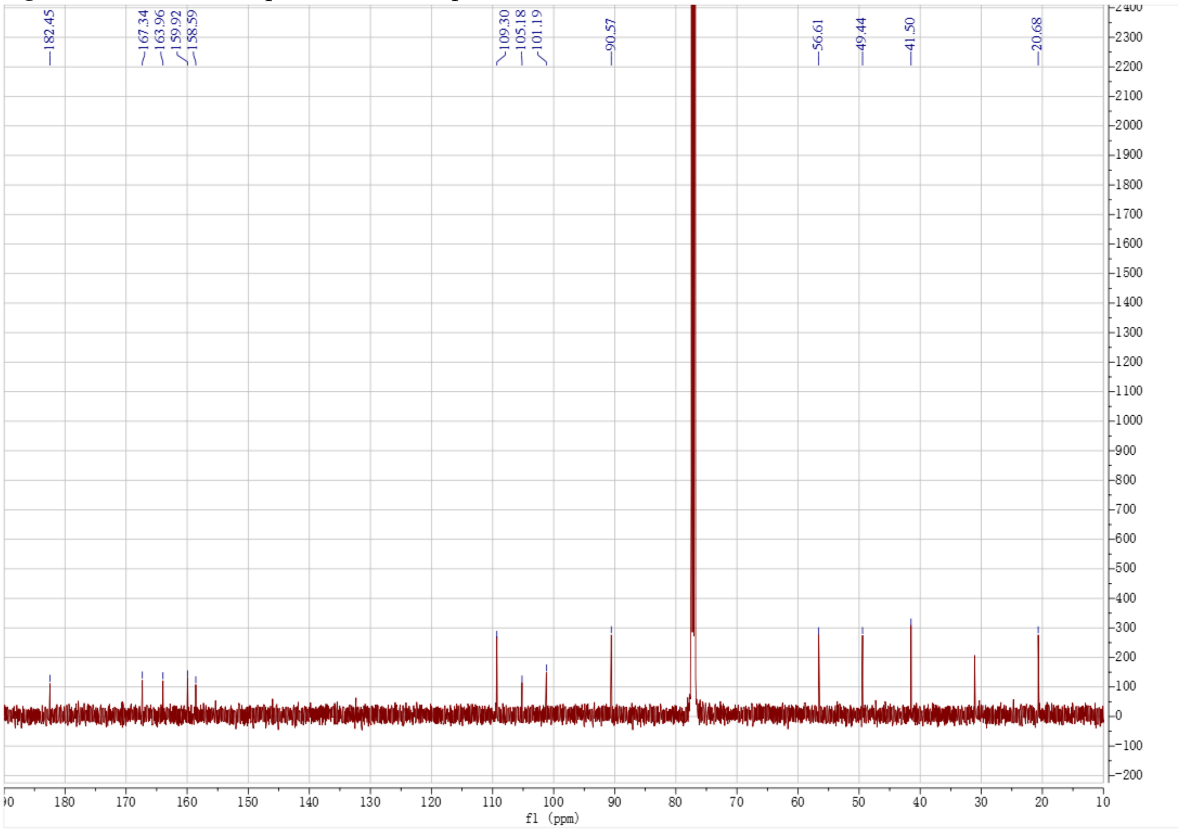

Figure S20. DEPT-135 spectrum of compound **5**.

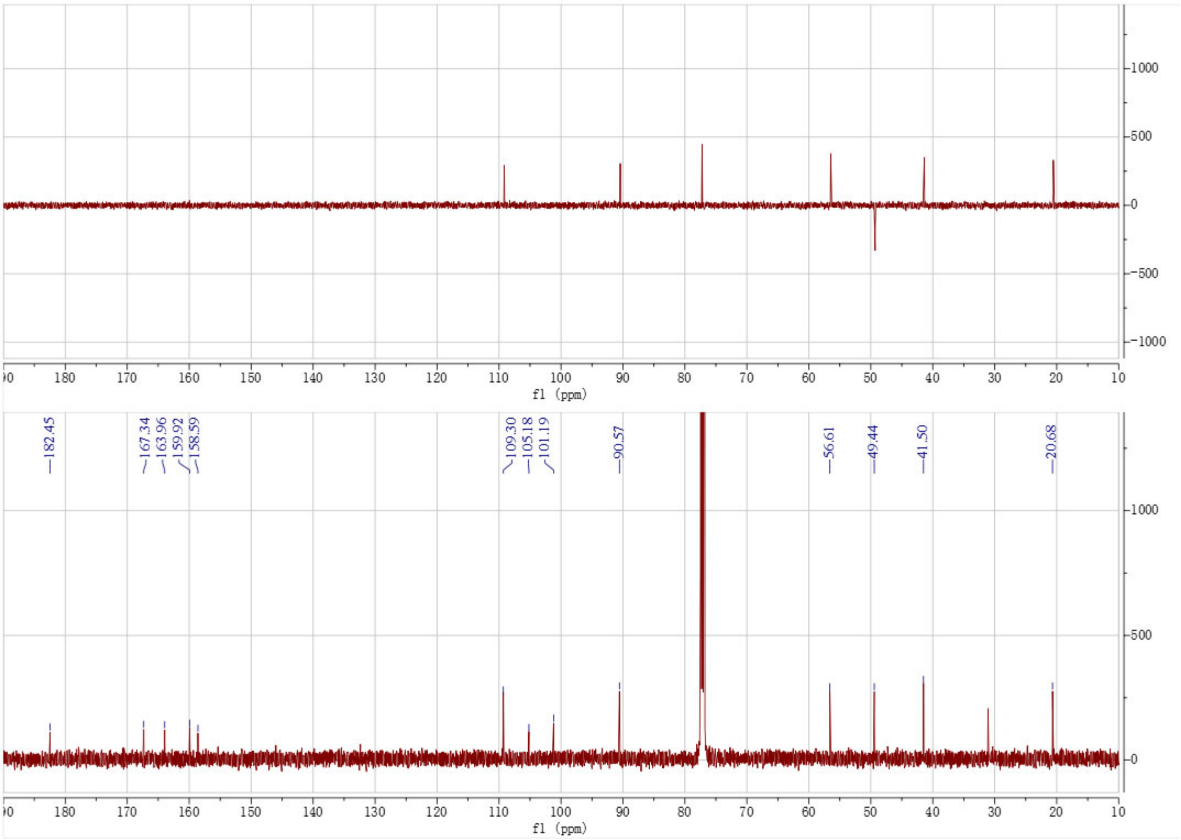

Figure S21. HSQC spectrum of compound **5**.

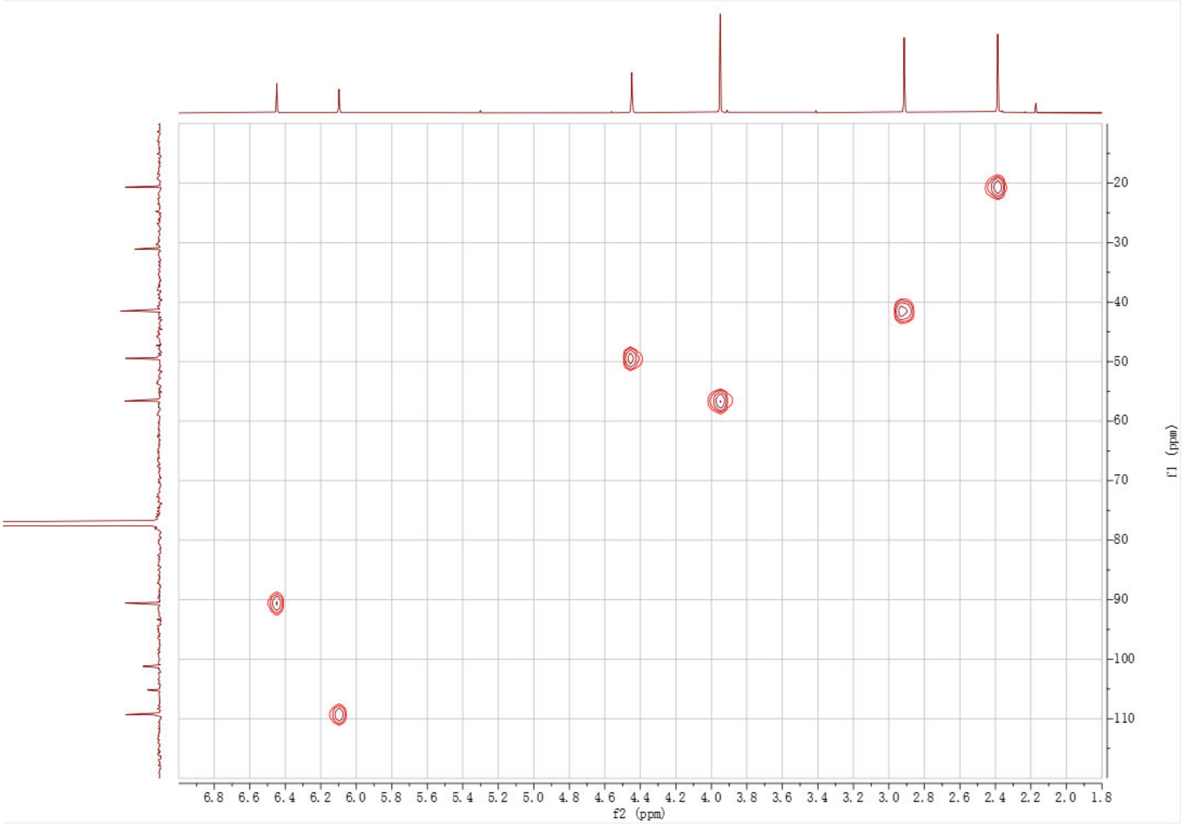

Figure S22. HMBC spectrum of compound **5**.

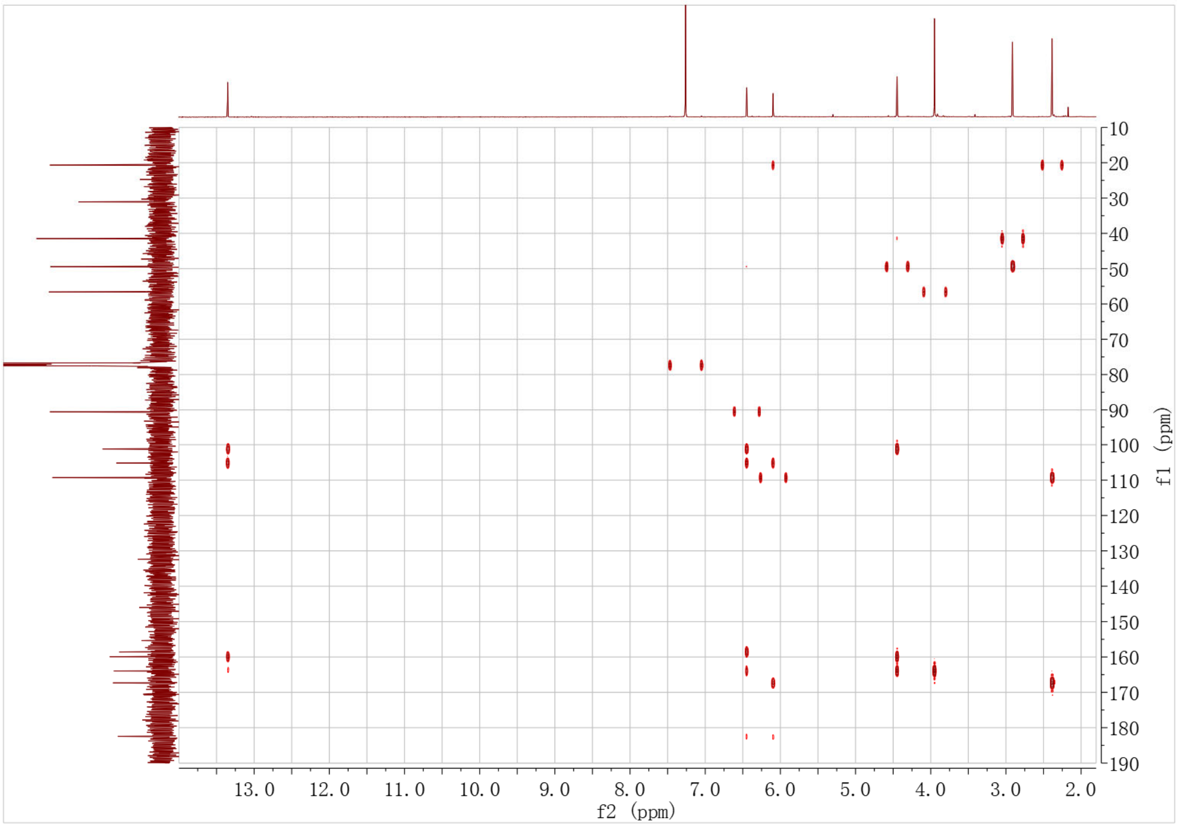

f2 (ppm)
f1 (ppm)
13.0
12.0
11.0
10.0
9.0
8.0
7.0
6.0
5.0
4.0
3.0
2.0
10
20
30
40
50
60
70
80
90
100
110
120
130
140
150
160
170
180
190

Figure S23. HRESIMS spectrum of compound **6**.

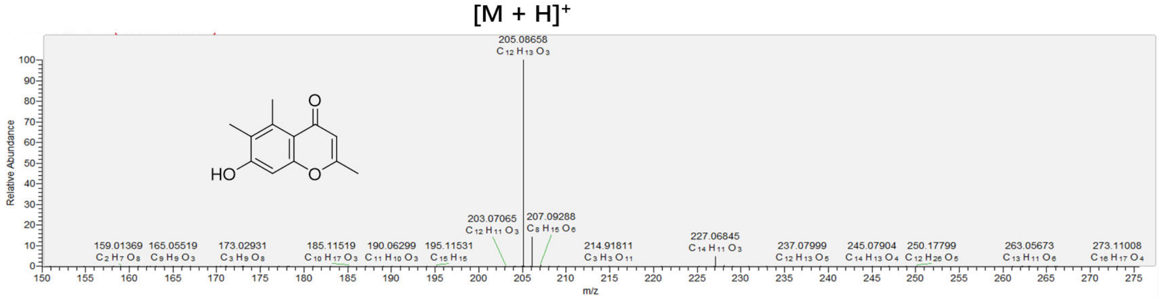

Figure S24. ^{1}H NMR (500 MHz, MeOD) spectrum of compound **6**.

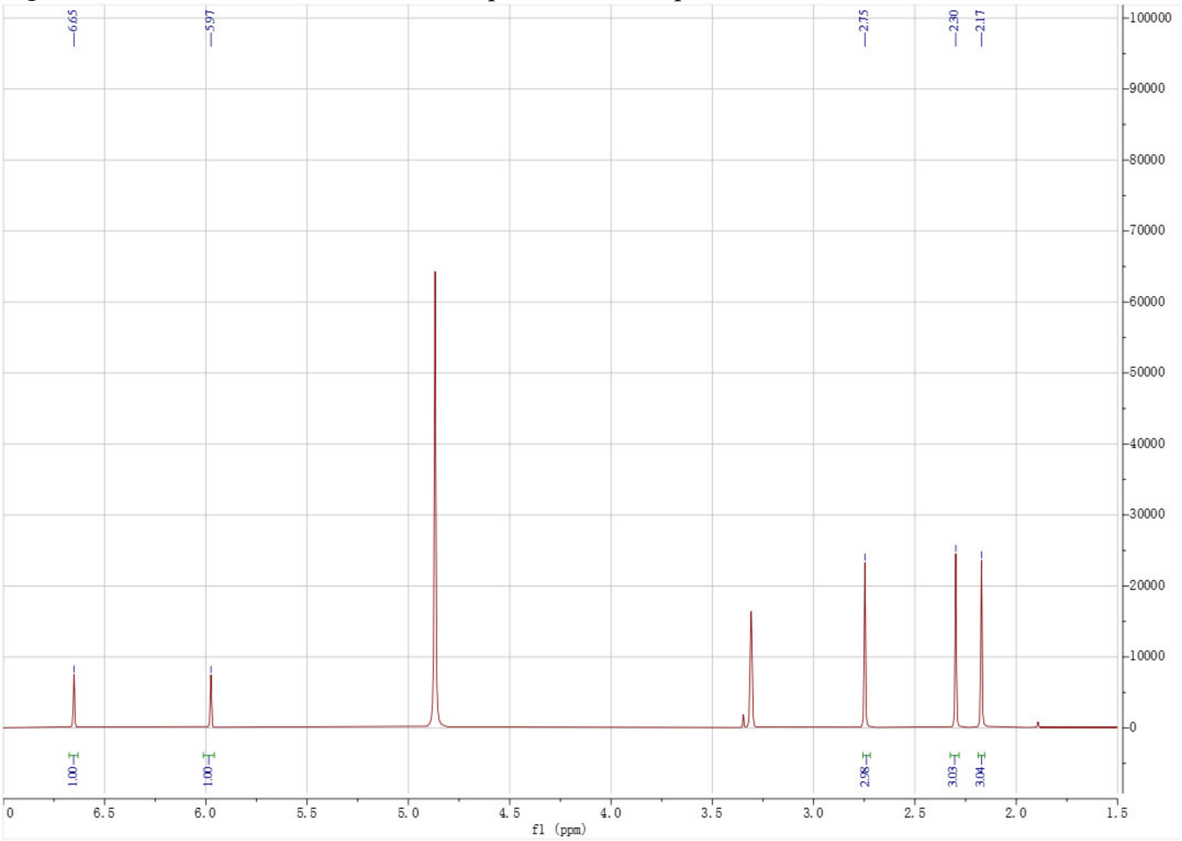

Figure S25. ^{13}C NMR spectrum of compound **6**.

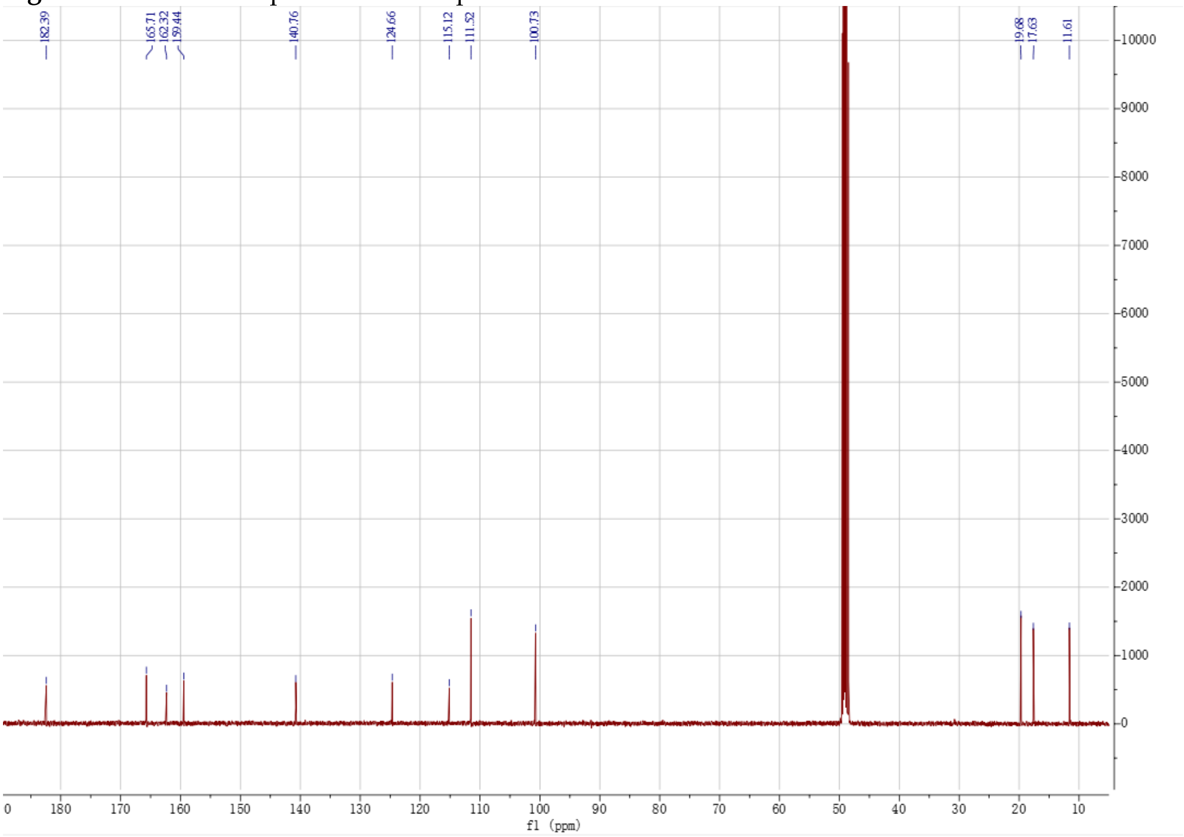

Figure S26. HSQC spectrum of compound **6**.

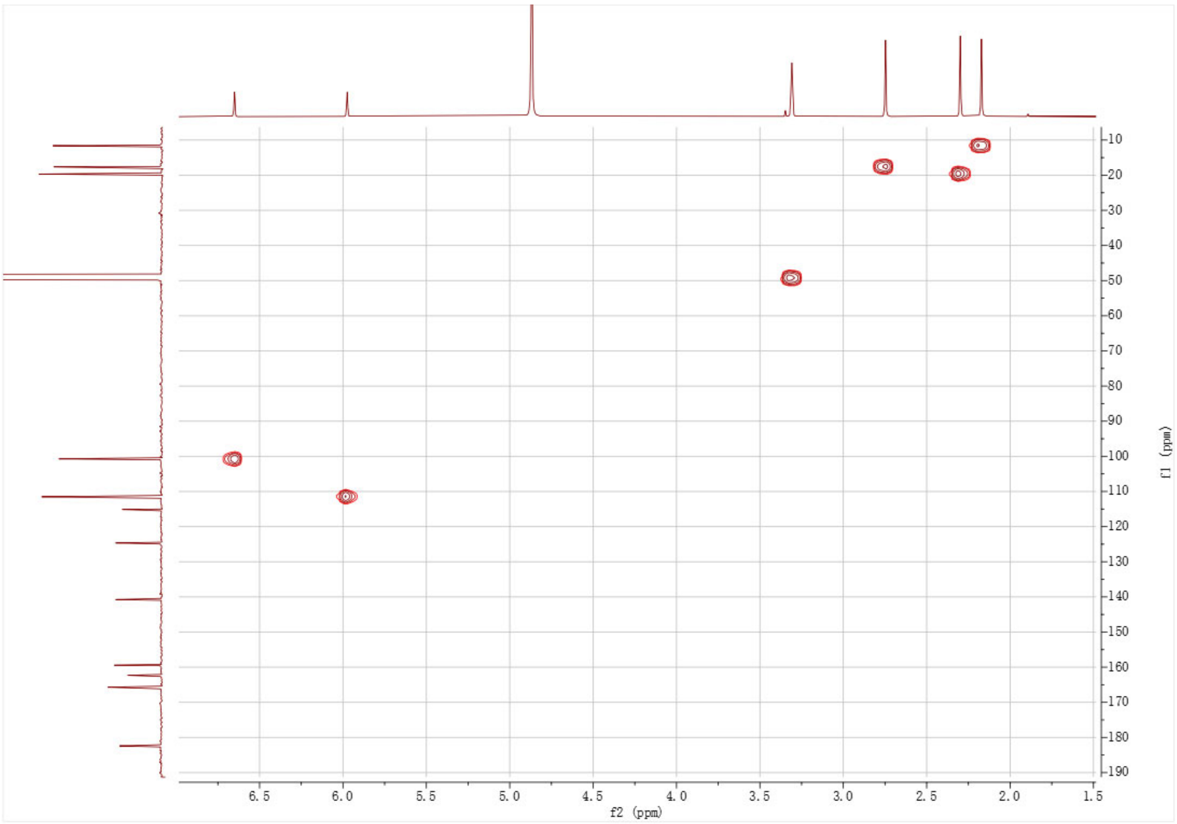

Figure S27. HMBC spectrum of compound **6**.

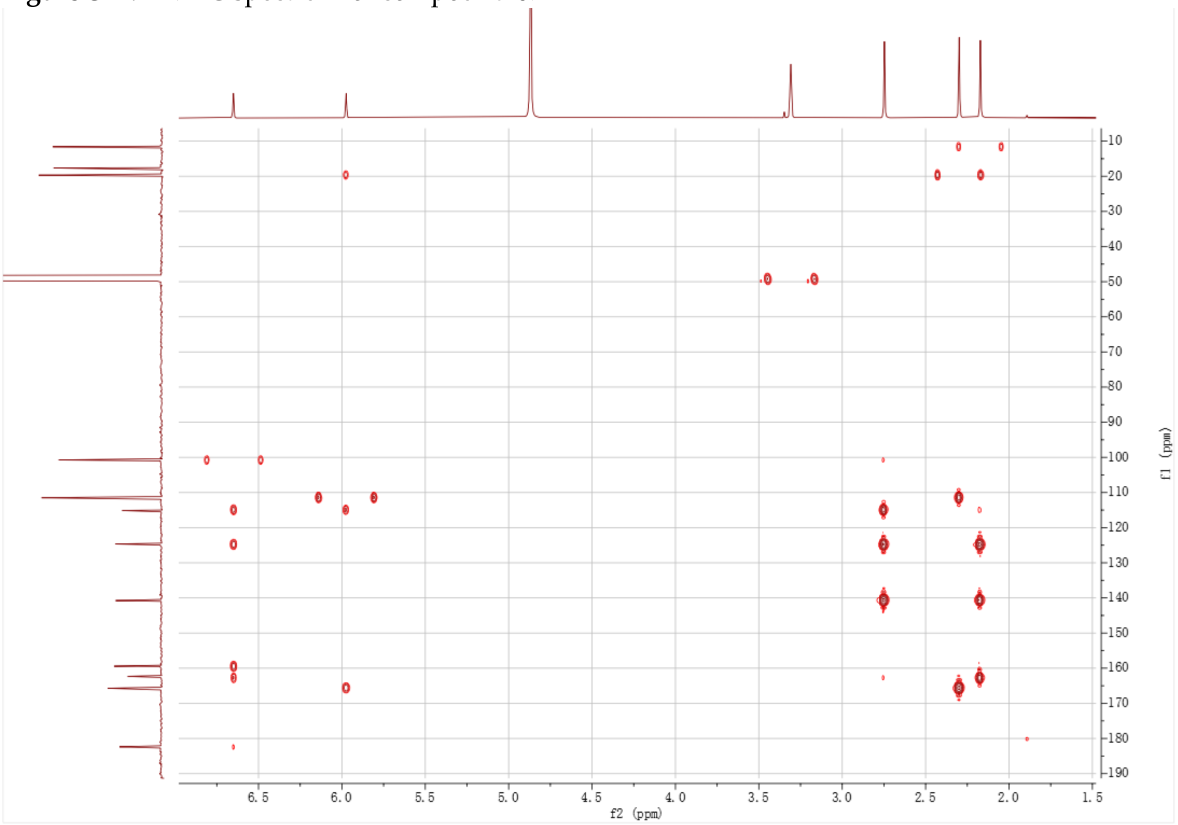

Figure S28. HRESIMS spectrum of compound **8**.

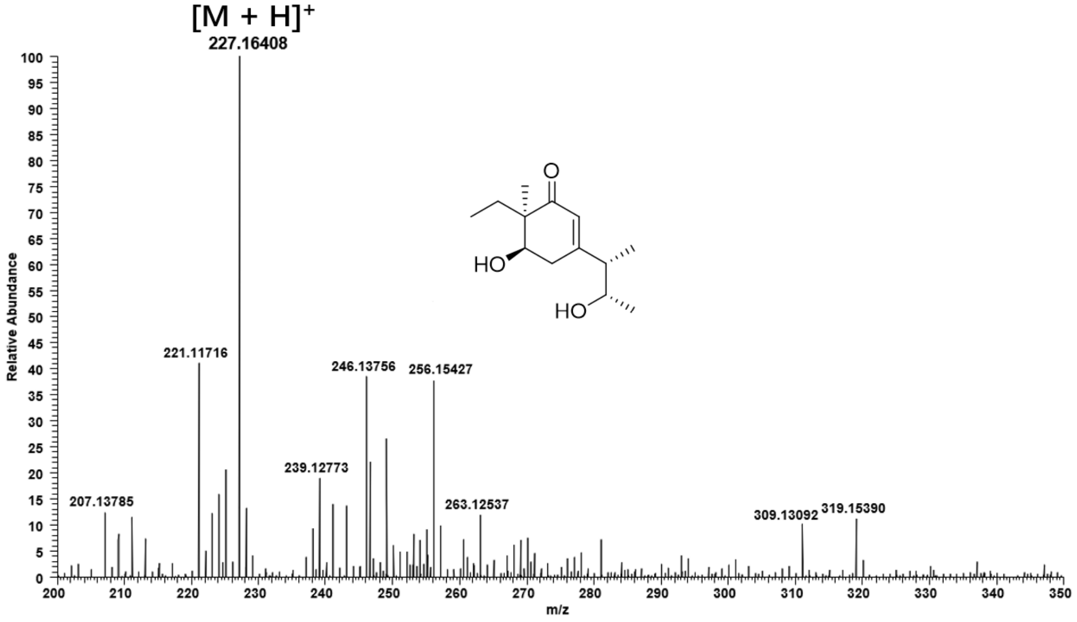

Figure S29. ^{1}H NMR (500 MHz, $CDCl_3$) spectrum of compound **8**.

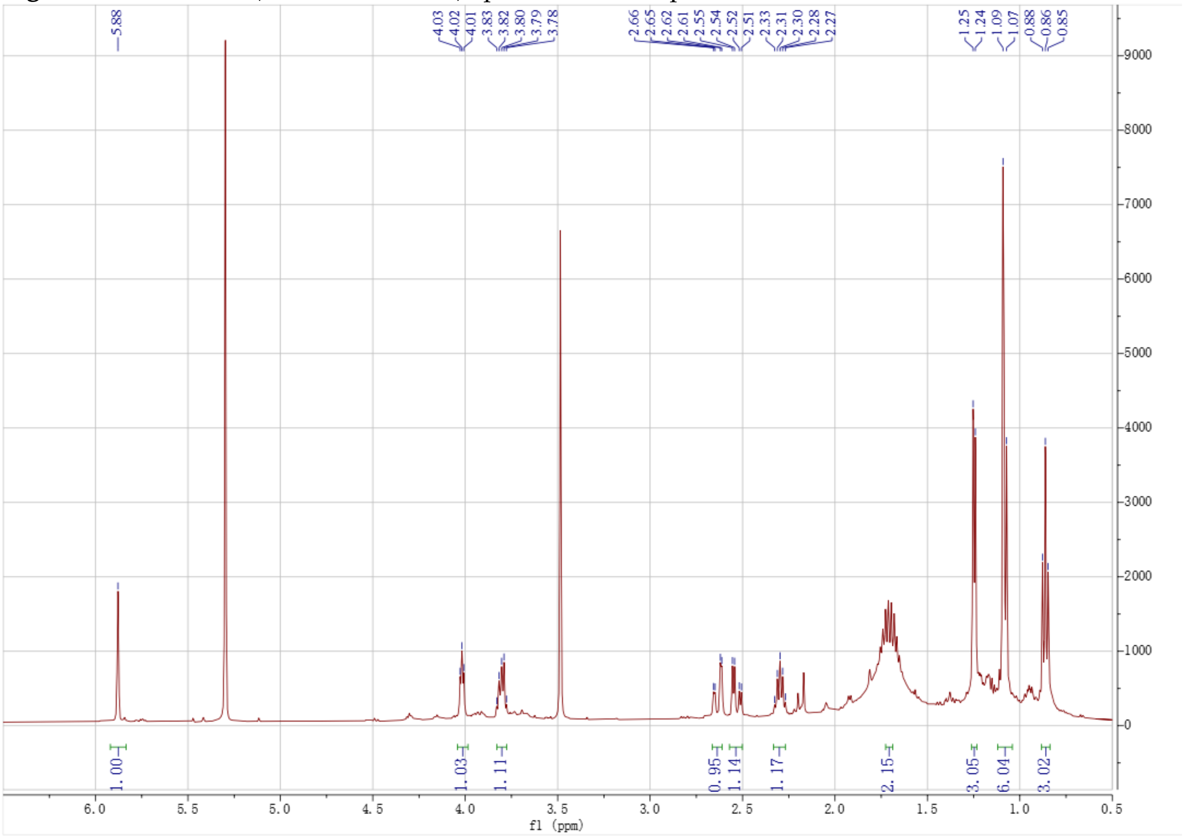

Figure S30. ^{13}C NMR spectrum of compound **8**.

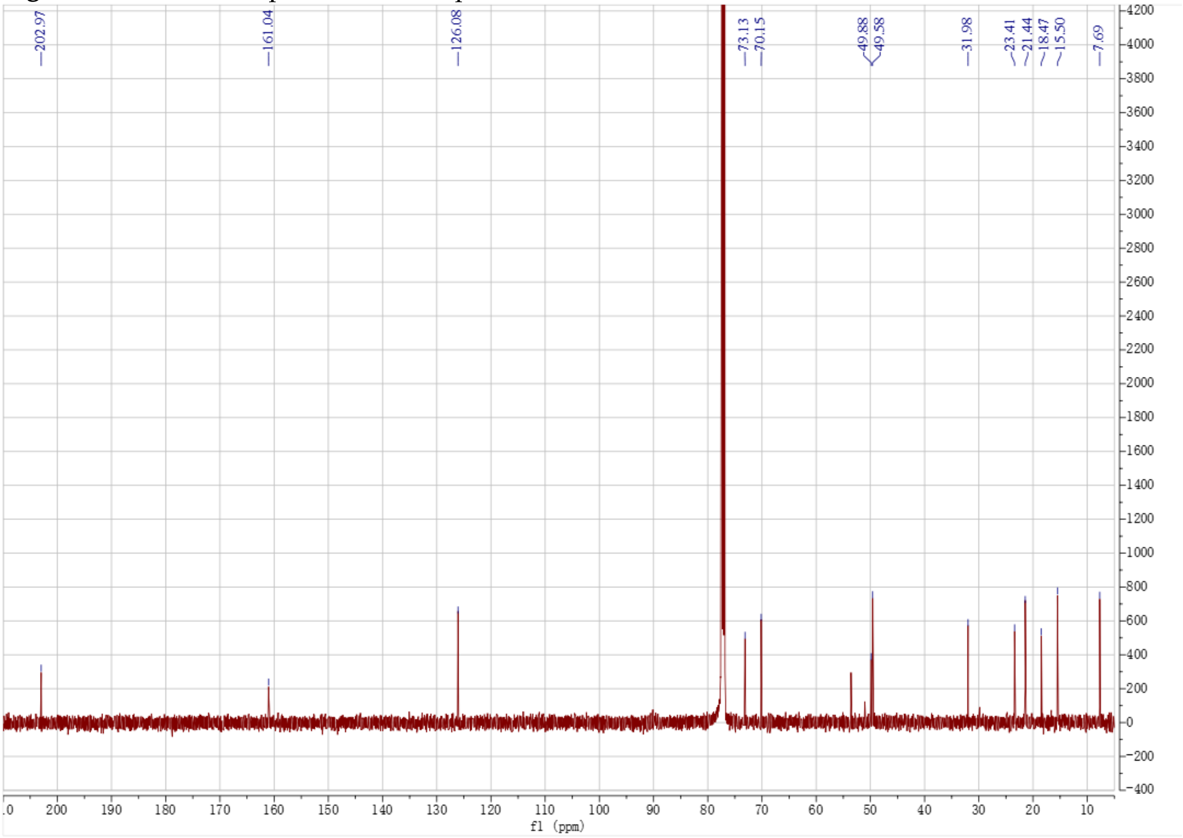

Figure S31. DEPT-135 spectrum of compound **8**.

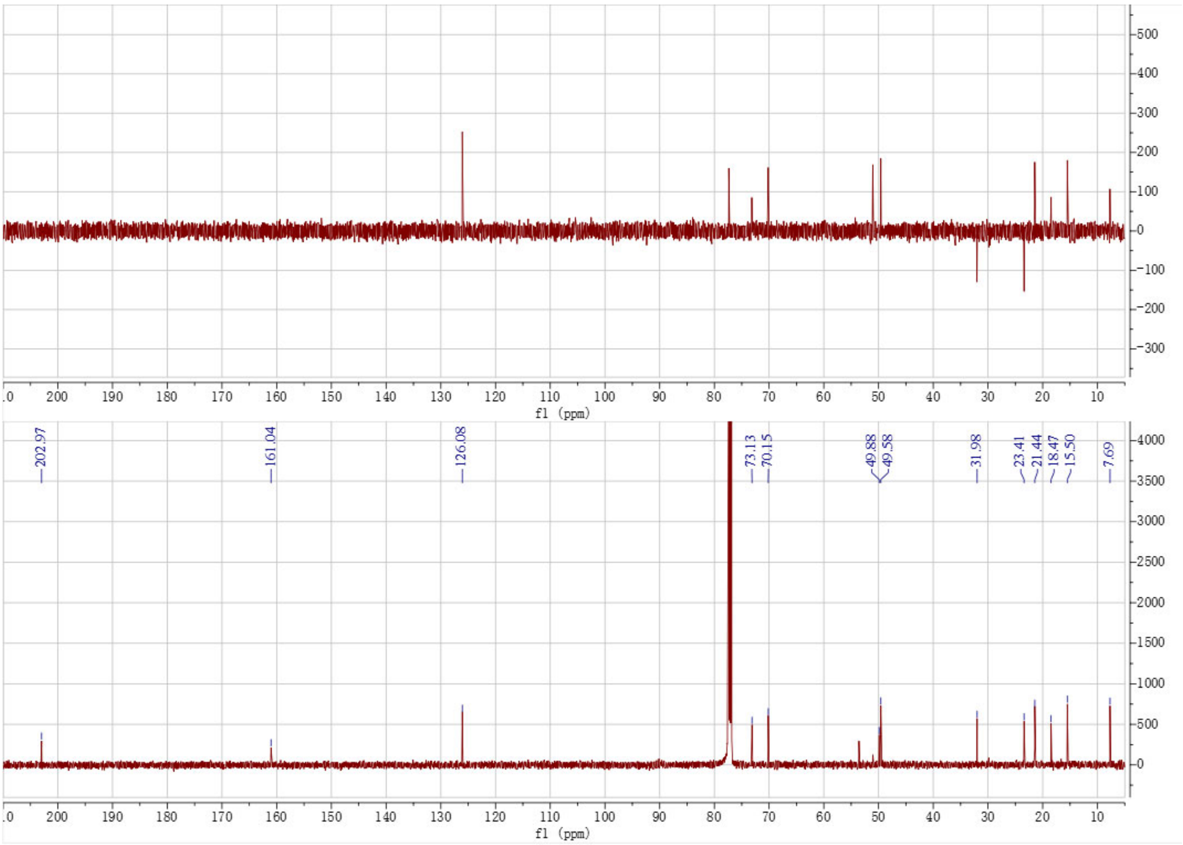

Figure S32. HSQC spectrum of compound **8**.

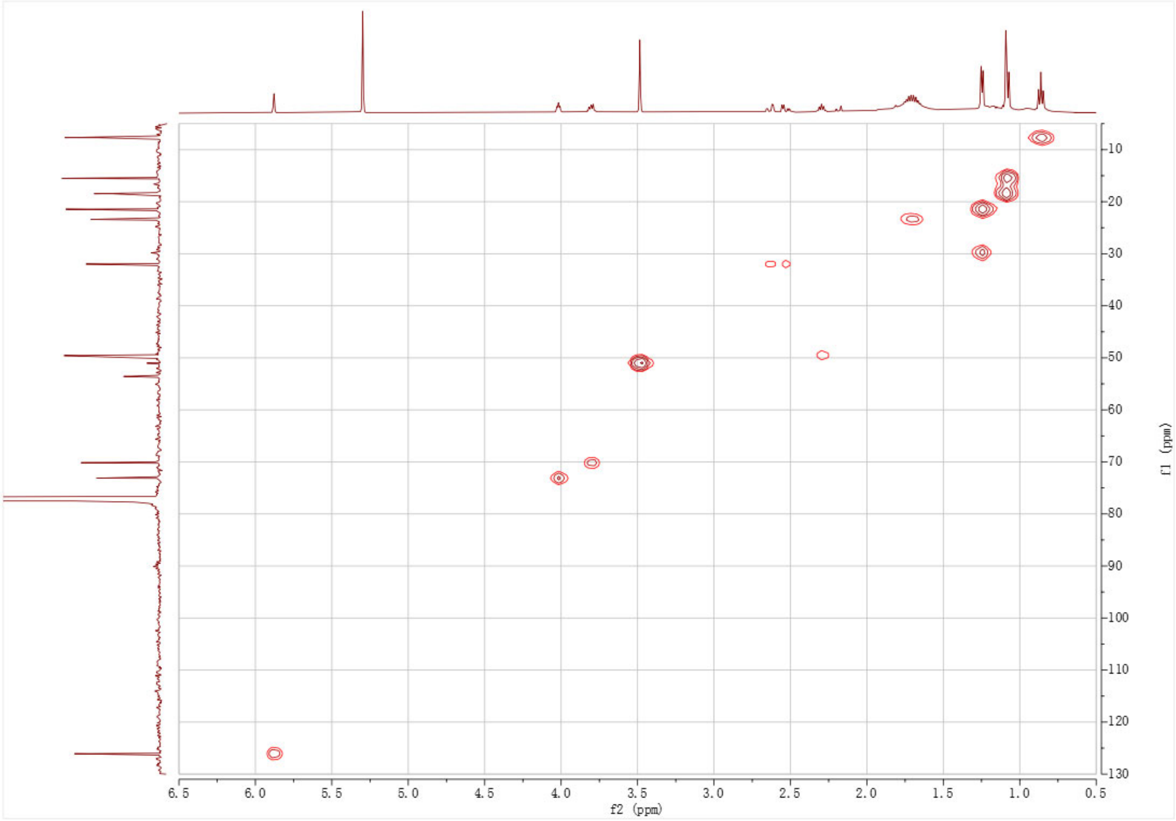

Figure S33. ^{1}H-^{1}H COSY spectrum of compound **8**.

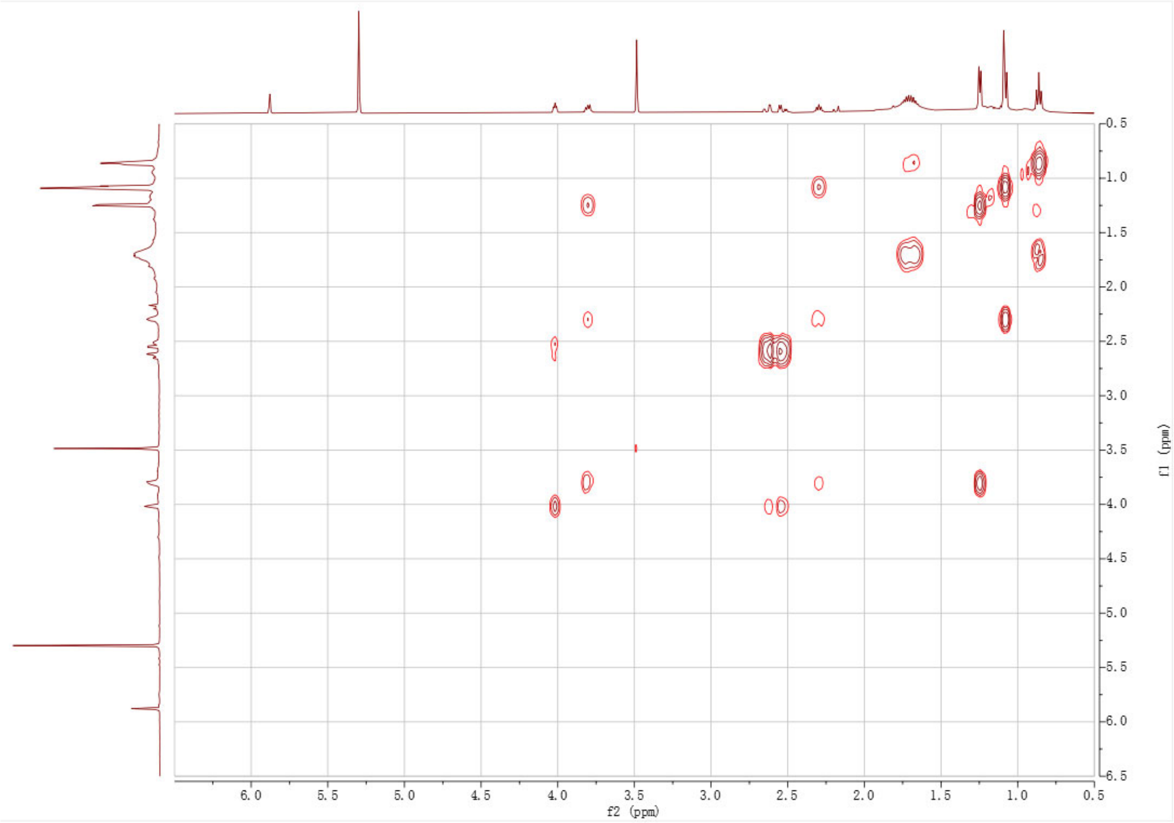

Figure S34. HMBC spectrum of compound **8**.

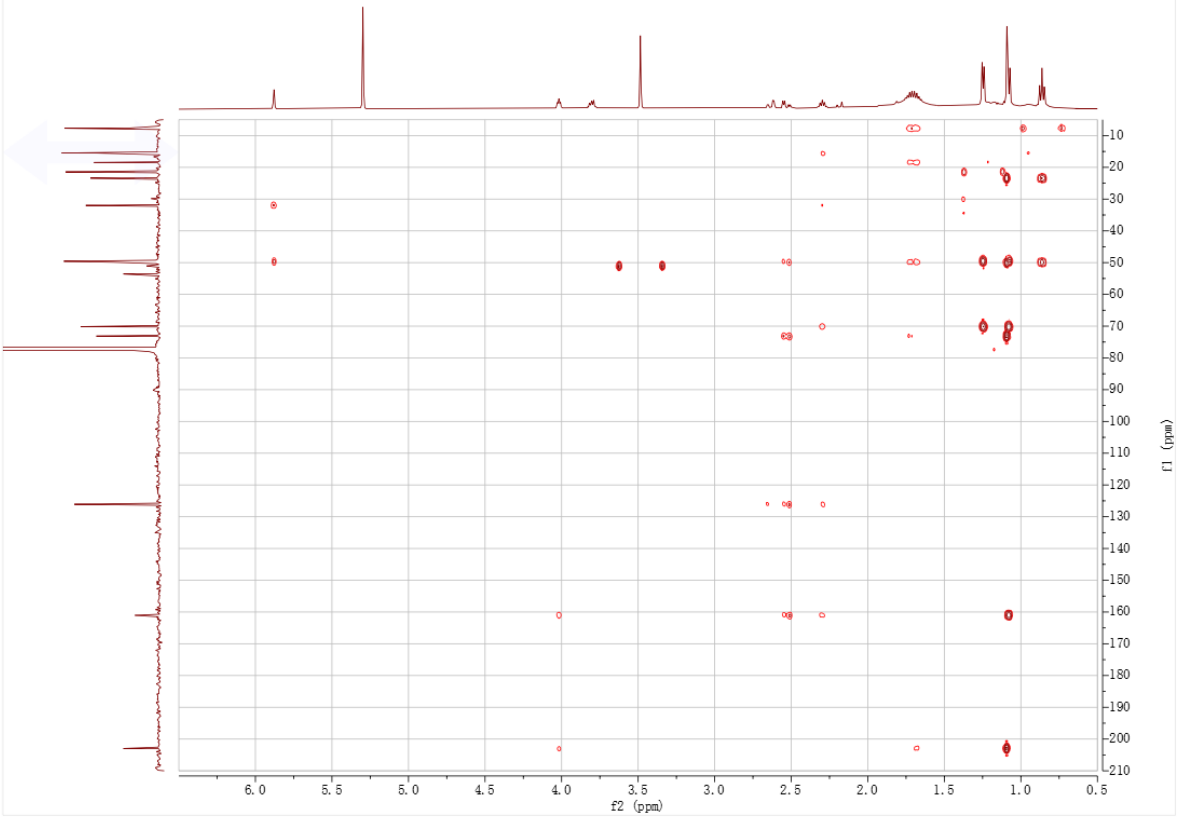

Figure S35. NOESY spectrum of compound **8**.

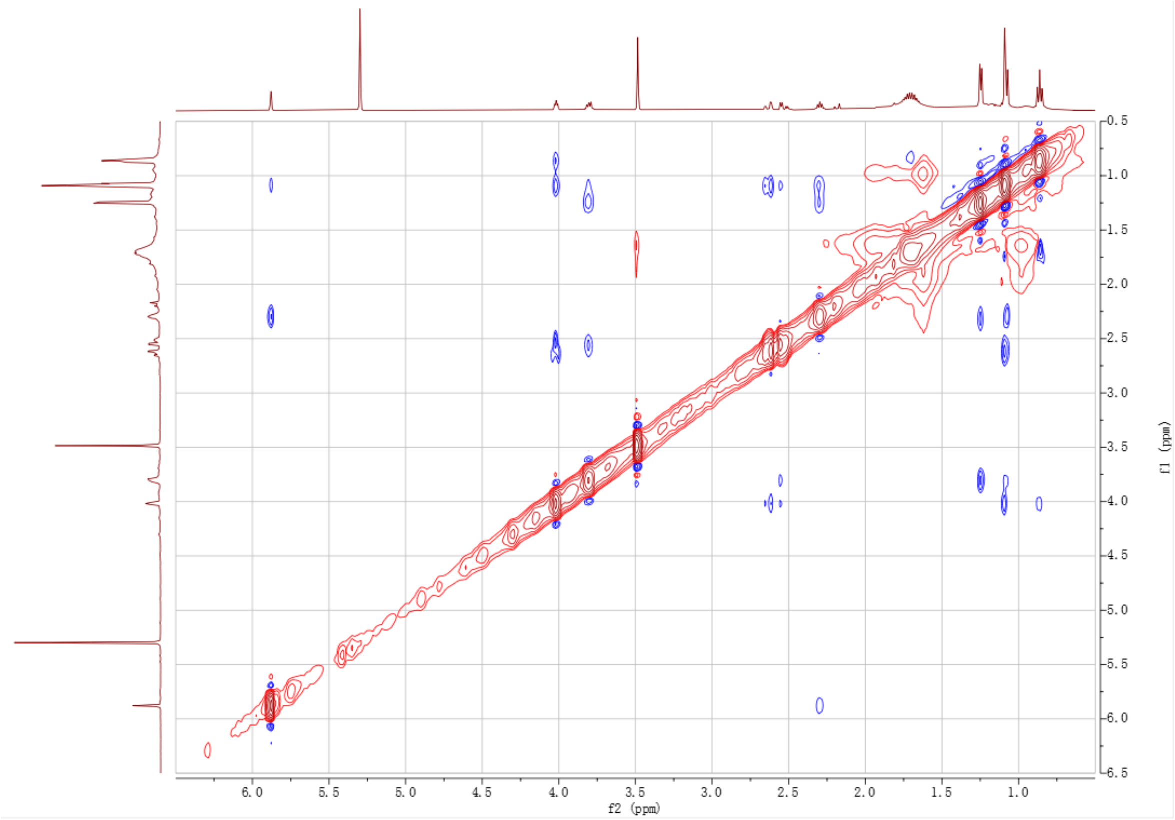

Figure S36. Optimized geometries of predominant conformers (weighting factors) for compound **1** at the B3LYP/6-311++g(d, p) level above 1% population.

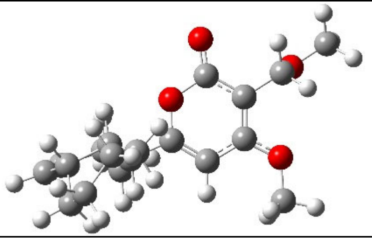	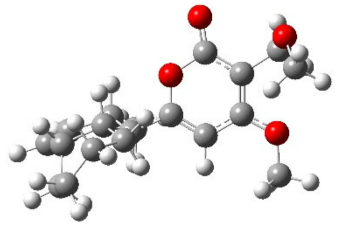	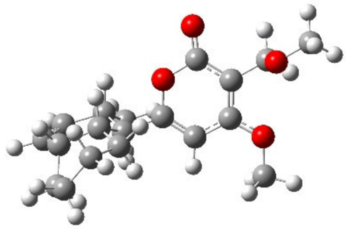
Energy: -1040.839559 a.u.<br>Conf.(39.7%)	Energy: -1040.838208 a.u.<br>Conf.(22.6%)	Energy: -1040.839589 a.u.<br>Conf.(37.6%)

Figure S37. Optimized geometries of predominant conformers (weighting factors) for compound **3** at the B3LYP/6-311++g(d, p) level above 1% population.

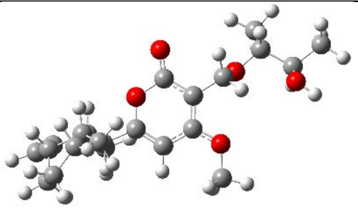	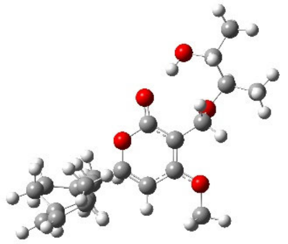	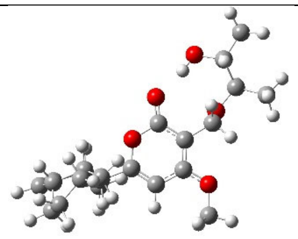
Energy: -1234.063677 a.u.<br>Conf.(1.0%)	Energy: -1234.066202 a.u.<br>Conf.(10.0%)	Energy: -1234.558504 a.u.<br>Conf.(88.0%)

Figure S38. Optimized geometries of predominant conformers (weighting factors) for compound **8** at the B3LYP/6-311++g(d, p) level above 1% population.

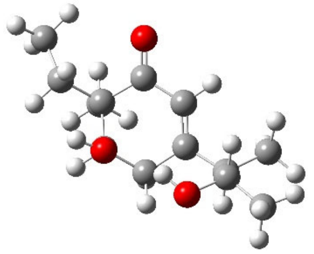	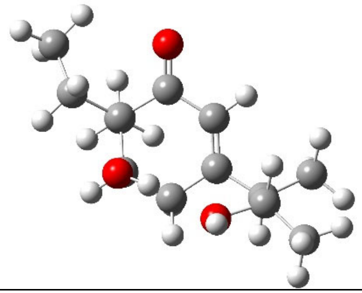
Energy: -734.52283 a.u.<br>Conf.(8.9%)	Energy: -734.52232 a.u.<br>Conf.(5.2%)
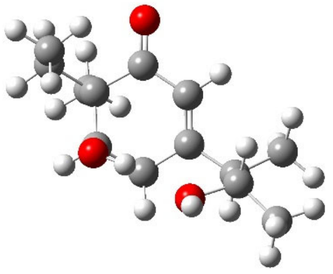	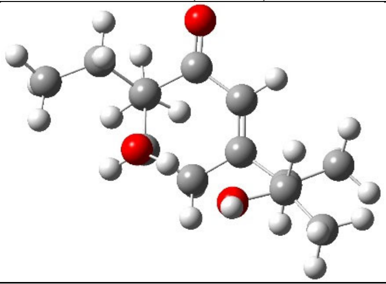
Energy: -734.52186 a.u.<br>Conf.(3.2%)	Energy: -734.52492 a.u.<br>Conf.(82.6%)

Figure S39. Regression analyses of experimental versus calculated ^{13}C NMR chemical shifts of compounds **1**, **3** and **8**.

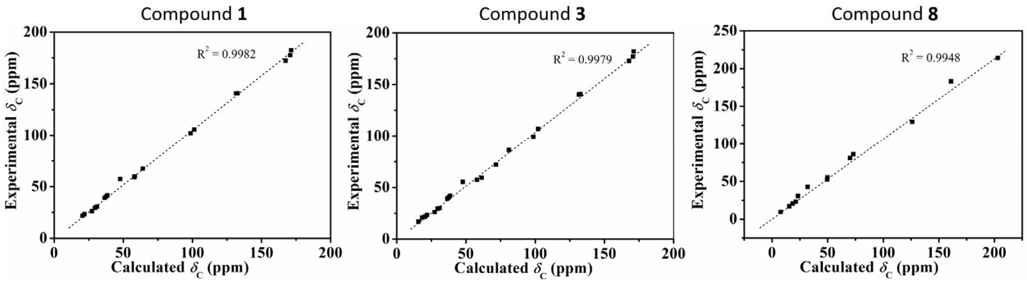

Figure S40. Herbicidal potential of **9** against the radicle growth of *Digitaria sanguinalis* seedlings.

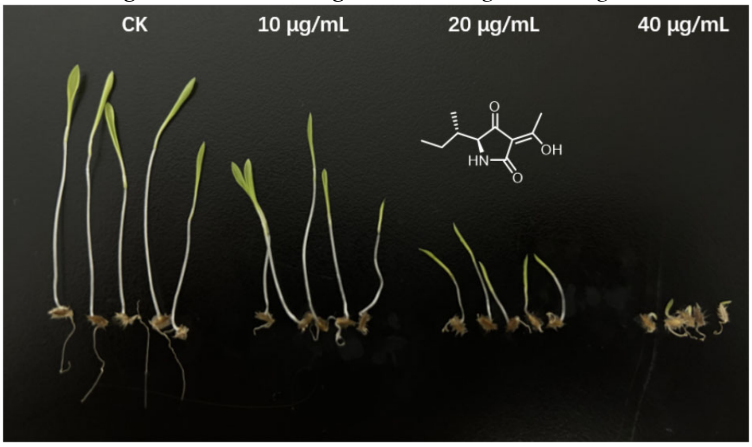

Table S1. Herbicidal spectrum of **9** against representative malignant weeds *D. sanguinalis*, *Portulaca oleracea*, and *Descurainia sophia.*

Weeds	Compounds	40 µg/mL	20	10
D. sanguinalis	**9**	88.6±2.1	84.1±1.5	73.2±1.9
	CK [a]	90.5±1.8	85.7±3.2	76.1±2.3
P. oleracea	**9**	71.9±2.2	62.6±1.4	n.d.
	CK [a]	89.2±2.9	81.3±1.7	n.d.
D. sophia	**9**	69.2±1.6	55.8±2.4	n.d.
	CK [b]	86.7±1.3	74.8±1.1	n.d.

[a,b] CK was chemical herbicide acetochlor [a] and tribenuron-methyl [b], respectively. n.d.: not detected.
